# Large‐Scale Production of Expandable Hepatoblast Organoids and Polarised Hepatocyte Organoids From hESCs Under 3D Static and Dynamic Suspension Conditions

**DOI:** 10.1111/cpr.70001

**Published:** 2025-02-08

**Authors:** Haibin Wu, Jue Wang, Shoupei Liu, Yiyu Wang, Xianglian Tang, Jinghe Xie, Ning Wang, Huanhuan Shan, Sen Chen, Xueyan Zhang, Weiping Zeng, Chuxin Chen, Yinjie Fu, Liangxue Lai, Yuyou Duan

**Affiliations:** ^1^ Laboratory of Stem Cells and Translational Medicine, Institute for Medical Research, The Second Affiliated Hospital, School of Medicine South China University of Technology Guangzhou China; ^2^ Laboratory of Stem Cells and Translational Medicine, Institute for Life Science, School of Medicine South China University of Technology Guangzhou China; ^3^ School of Biomedical Sciences and Engineering South China University of Technology, Guangzhou International Campus Guangzhou China; ^4^ Key Laboratory of Regenerative Biology, South China Institute for Stem Cell, Biology and Regenerative Medicine, Guangzhou Institutes of Biomedicine and Health Chinese Academy of Sciences Guangzhou China; ^5^ National Engineering Research Center for Tissue Restoration and Reconstruction South China University of Technology Guangzhou China; ^6^ The Innovation Centre of Ministry of Education for Development and Diseases, The Second Affiliated Hospital, School of Medicine South China University of Technology Guangzhou China

**Keywords:** 3D suspension culture, bioreactor, extracellular matrix, hepatoblast organoid, human embryonic stem cells, polarised hepatocyte organoids

## Abstract

To date, generating viable and functional hepatocytes in large scale remains challenge. By employing 3D suspension condition with the support of low concentration Matrigel, a novel culture system was developed to generate expandable hepatoblast organoids (HB‐orgs) and mature polarised hepatocyte organoids (P‐hep‐orgs) from human embryonic stem cells (hESCs) in both dishes and bioreactors. scRNA‐seq and functional assays were used to characterise HB‐orgs and P‐hep‐orgs. hESC‐derived HB‐orgs could proliferate at least for 15 passages, leading to 10^12^ in total cells in 4 weeks. P‐hep‐orgs differentiated from HB‐orgs displayed characteristics of mature hepatocytes with polarisation. Moreover, single‐cell RNA sequencing exhibited that over 40% of cells in P‐hep‐orgs were highly fidelity with human primary hepatocytes. Eventually, large‐scale production of P‐hep‐orgs could be generated from massively expanded HB‐orgs within 1 week with similar number in bioreactors, which were achieved by the enhancements in energy metabolism contribute to the expansion of HB‐orgs and maturation of P‐hep‐orgs in bioreactors. By providing a cost‐efficient and robust platform, our study represents a significant step toward manufacturing large‐scale functioning hESC‐derived hepatocytes for cell‐based therapeutics, disease modelling, pharmacology and toxicology studies.

## Graphical abstract

Under 3D suspension culture condition, the expandable hepatoblast organoids and mature polarised hepatocyte organoids were established by low concentration of Matrigel from human embryonic stem cells, which recapitulated the gene expression signatures and functions of primary human hepatoblast and hepatocytes. The dynamic culture system by using bioreactors further enlarged the scale of hepatocyte organoid culture.

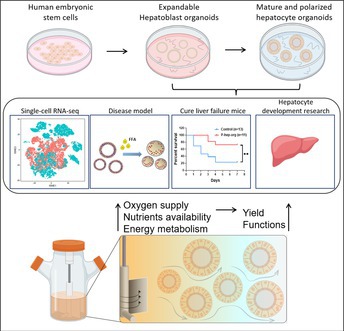

## 1 Introduction

The liver is responsible for metabolism, detoxification, bile secretion and other functions [1]. Nowadays, orthotopic liver transplantation is only the optimal treatment for patients with acute liver failure (ALF) or the end stage of liver diseases [2], and hepatocyte transplantation and biological artificial liver system are also alternative treatments. Although the liver has the outstanding ability to regenerate [3], primary hepatocytes are difficult to be cultured and maintained in vitro [4]. To address this problem, modifications of primary hepatocytes (e.g. long‐term culture of primary human hepatocytes [PHH] by chemical molecules treatment [5, 6, 7]) and alternative cell sources (e.g. conversion of fibroblasts into hepatocytes [8]) have been extensively studied in recent years. However, it is still challenging to fulfil clinical requirements because of the poor maturity and function as well as the difficulty to scale up hepatocytes in vitro [9].

Hepatocytes exhibit a multi‐polarised state under in vivo physiological environment [10], the polarisation of hepatocytes is indispensable for the secretion and uptake of the bile and other components. However, currently, polarised hepatocytes only could be cultured through collagen sandwich culture model [11] or transwell culture method [12] in 2D culture systems, which is very inconvenient and incapable to scale up. Moreover, 2D culture system occupies large spaces and consumes huge efforts for scaling up production, for example, by using a multilayer cell factory. 3D culture system for hepatocytes has been shown to be more advantageous in mimicking the in vivo environment and more favourable to obtain hepatocytes with higher functionality than 2D culture systems [13]. Owing to the limitations of PHHs in availability and genetic heterogenicity among different donors, it is still challenging to apply it into pharmaceutical industry and clinical applications [14]. Human pluripotent stem cells (hPSCs), including human embryonic stem cells (hESCs) and human‐induced pluripotent stem cells (hiPSCs), have great potential as a robust cell source in regenerative medicine due to their capacity for self‐renewal and multi‐lineage differentiation potential [15, 16, 17]. Furthermore, hPSCs are more convenient to be cultured and expanded under 3D conditions in large scale without losing pluripotency [18, 19]. Hepatocyte organoids have been established from hPSCs and progenitor cells isolated from liver. These organoids hold great potential for applications in disease modelling, cell transplantation therapy and drug testing [20, 21]. Previous studies have established defined and scalable methods for generating hepatocyte‐like cells from hPSCs under both 2D [22] and 3D conditions [23, 24], but the resulting hepatocyte‐like cells often exhibit limited functional maturity. Bioreactor‐based scalable culture methods have also been explored for organoids derived from hPSCs, such as retinal [25] and kidney [26] organoids, as well as liver ductal cells from primary human liver tissue [27]. However, current protocols for scalable and reproducible methods remain a challenge, for example, hepatocyte organoids culture are restrictively dependent on hanging‐drop Matrigel or other synthetic extracellular matrix with high concentration [28, 29], limiting the practicability of the mass production of organoid in large scale under 3D suspension culture systems with bioreactors.

In this study, we established a new system to generate mature polarised hepatocyte organoids (P‐hep‐orgs) in large scale from expandable and storable hESC‐derived hepatoblast organoids (HB‐orgs) under 3D dynamic suspension culture systems through developed culture mediums with the support of Matrigel at low concentration in bioreactors. Our HB‐orgs manifested the expansion potential for at least 15 passages (to date) with chromosome stability and bipotency. Moreover, the polarised hepatocyte organoids (P‐hep‐orgs) could be further generated from HB‐orgs around 1 week, exhibited mature hepatocyte phenotypes and improved liver functions compared with non‐polarised hepatic spheres. Single‐cell RNA sequencing (scRNA‐seq) analysis for HB‐orgs and P‐hep‐orgs was used to characterise and compare with human fetal and primary hepatocytes. Finally, the applications of our P‐hep‐orgs for disease modelling, and the rescue of ALF mice, were also investigated.

## 2 Results

### 2.1 Generation of Expandable HB‐Orgs From hESCs Under 3D Suspension Culture

**FIGURE 1:**
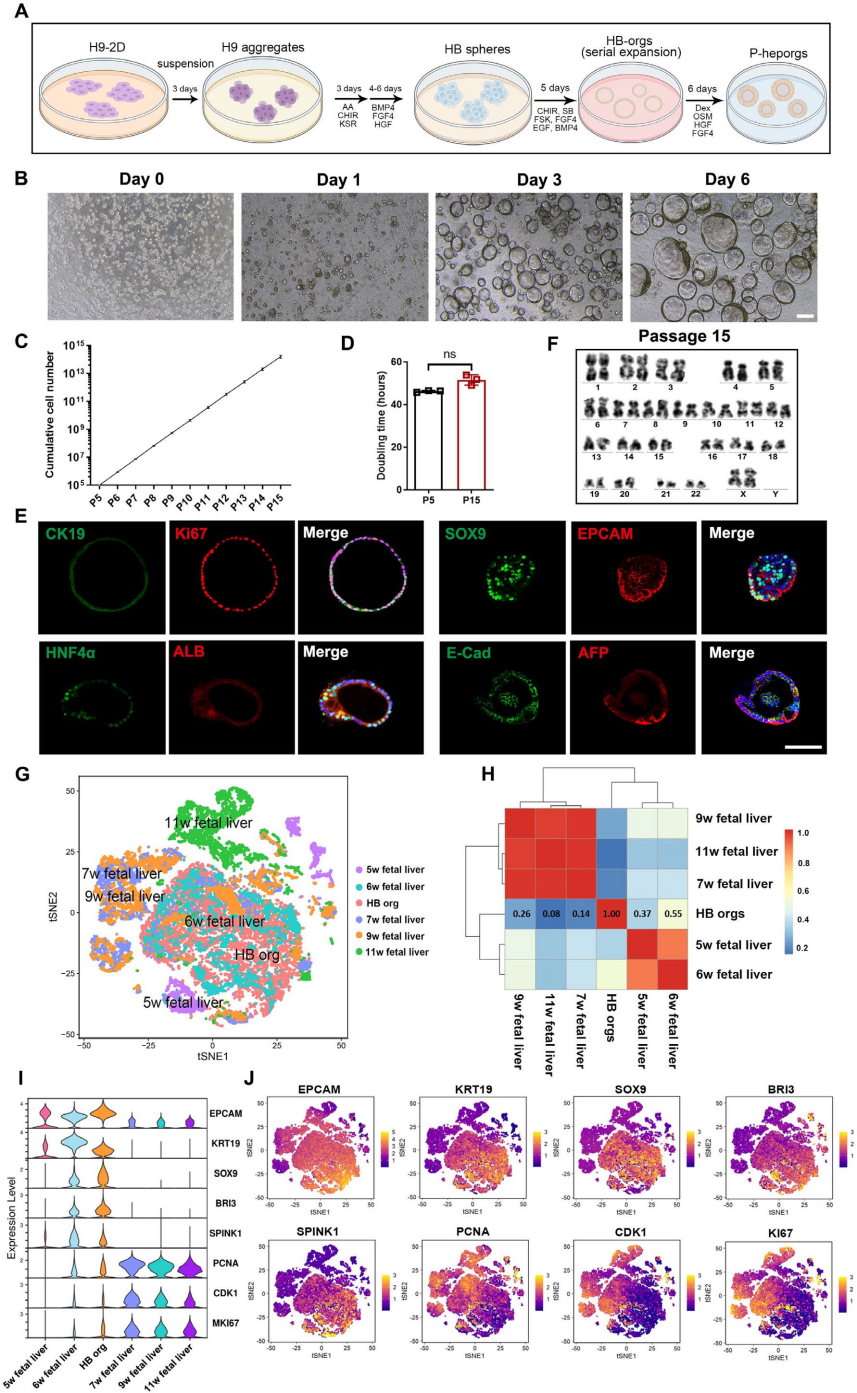
Generation and characterisation of HB‐orgs under 3D suspension culture. (A) Schematic illustration of differentiating HB‐orgs and P‐hep‐orgs from hESCs in 3D suspension culture. (B) Representative morphologies of HB‐orgs at different days post‐passage. Scale bar = 100 μm. (C) Cumulative cell count of HB‐orgs from Passages 5 to 15. (D) Doubling times of HB‐orgs at passages 5 and 15. (E) Co‐immunostaining for CK19 and Ki67, HNF4α and ALB, SOX9 and EPCAM, E‐cad and AFP in HB‐orgs. Nuclei were stained with DAPI. Scale bar = 100 μm. (F) Representative karyotype analysis of HB‐orgs at passage 15 by G‐banding. (G) t‐SNE clustering of integrated single‐cell transcriptomes from HB‐org (*n* = 6880) and human fetal livers (5–11 weeks, *n* = 14,526), coloured by original source. (H) Correlation heatmap of integrated analysis from (G). (I) Violin plot representation of hepatoblast genes in HB‐orgs and human fetal liver cells. (J) t‐SNE representation of hepatoblast and cell proliferation genes in integrated single‐cell transcriptomes of HB‐orgs and human fetal livers. Results were presented as mean ± SD.

We developed a differentiation protocol based on our previous methods [30] to generate HB spheres from hESCs, H9 cells in 3D suspension culture system (Figure 1A). In this approach, H9 cell aggregates were first cultured in mTeSR1 medium for 3 days (Figure S1). The aggregates were then induced to differentiate into definitive endoderm spheres (Figure S2A–D), followed by further differentiation into HB spheres (Figure S3A). These HB spheres exhibited high expression of key HB markers, including AFP, EPCAM, FOXA2, SOX9, TBX3 and HNF4α (Figure S3B–D), confirming the successful generation of pure HB spheres from H9 cells under 3D suspension condition.

hESCs exhibited a great proliferation capacity as aggregates in mTeSR1 medium under 3D suspension culture. To culture HB spheres in vitro, we developed a defined HB pre‐expansion medium containing chemicals and growth factors that support HB proliferation [31]. It has been shown that extracellular matrix was benefited for the viability and function of hepatocytes [32], thus, after comparing different concentrations of Matrigel (Figure S4A), and considering the feasibility of large‐scale generation of hepatocytes, 5% (vol/vol) Matrigel was selected to culture HB spheres. HB spheres formed organoid structures (HB‐orgs) and expanded efficiently through multiple passages, with an increase in cell numbers under the support of Matrigel (Figure 1B). We then optimised HB pre‐expansion medium by systematically removing chemicals and growth factors one by one. Morphological observations (Figure S4B) and organoid formation rates revealed that CHIR99021, SB431542 and FSK were crucial for HB‐orgs expansion (Figure S4C). Interestingly, the percentages of Ki67^high^ cells increased significantly in the absence of Y27632 and HGF, indicating these factors were unnecessary for HB‐orgs proliferation (Figure S4D,E). Ultimately, the key components of our HB‐expansion medium were identified as CHIR99021, SB431542, FSK, BMP4, FGF4 and EGF. Under these conditions, HB‐orgs were expanded 6–8 folds per passage every 5–6 days, for at least 15 passages (Figure 1C). The doubling time was approximately 45 h (Figure 1D), and HB markers SOX9, EPCAM, HNF4A, CK19, AFP, ALB, E‐cad and the proliferation marker Ki67 were highly expressed (Figure 1E). Karyotype analysis confirmed chromosomal stability at passage 15 (Figure 1F), and the expression of HB‐specific markers was consistent between passages 5 and 15 (Figure S5A), demonstrating the long‐term stability of our HB‐orgs under 3D suspension culture.

scRNA‐seq of HB‐orgs (*n* = 6880) was performed and compared with human fetal livers at 5–11 weeks (*n* = 14,526) [33]. Integrated analyses showed that HB‐orgs closely resembled human fetal liver at 6 weeks (Figure 1G,H), and a large number of cells highly expressed HB genes EPCAM, KRT19, SOX9, BRI3, SPINK1, PCNA [34], along with proliferation genes CDK1 and Ki67 (Figure 1I,J), confirming HB‐like nature of HB‐orgs.

HB‐orgs retained their key characteristics and expansion capacity after being thawed from the cryopreservation (Figure S5B,C). To further test the stability and reproducibility of our culture system, we used another hESC line (H1 cells) and a hiPSC line (UH10 cells), both of which were successfully differentiated into HB‐orgs (Figure S5D,E). These results confirmed that our hESC‐derived HB‐orgs are not only expandable and storable but also a robust cell source for large‐scale hepatocyte production.

### 2.2 Differentiation of HB‐Orgs Towards Mature Polarised Hepatocyte Organoids (P‐Hep‐Orgs)

**FIGURE 2:**
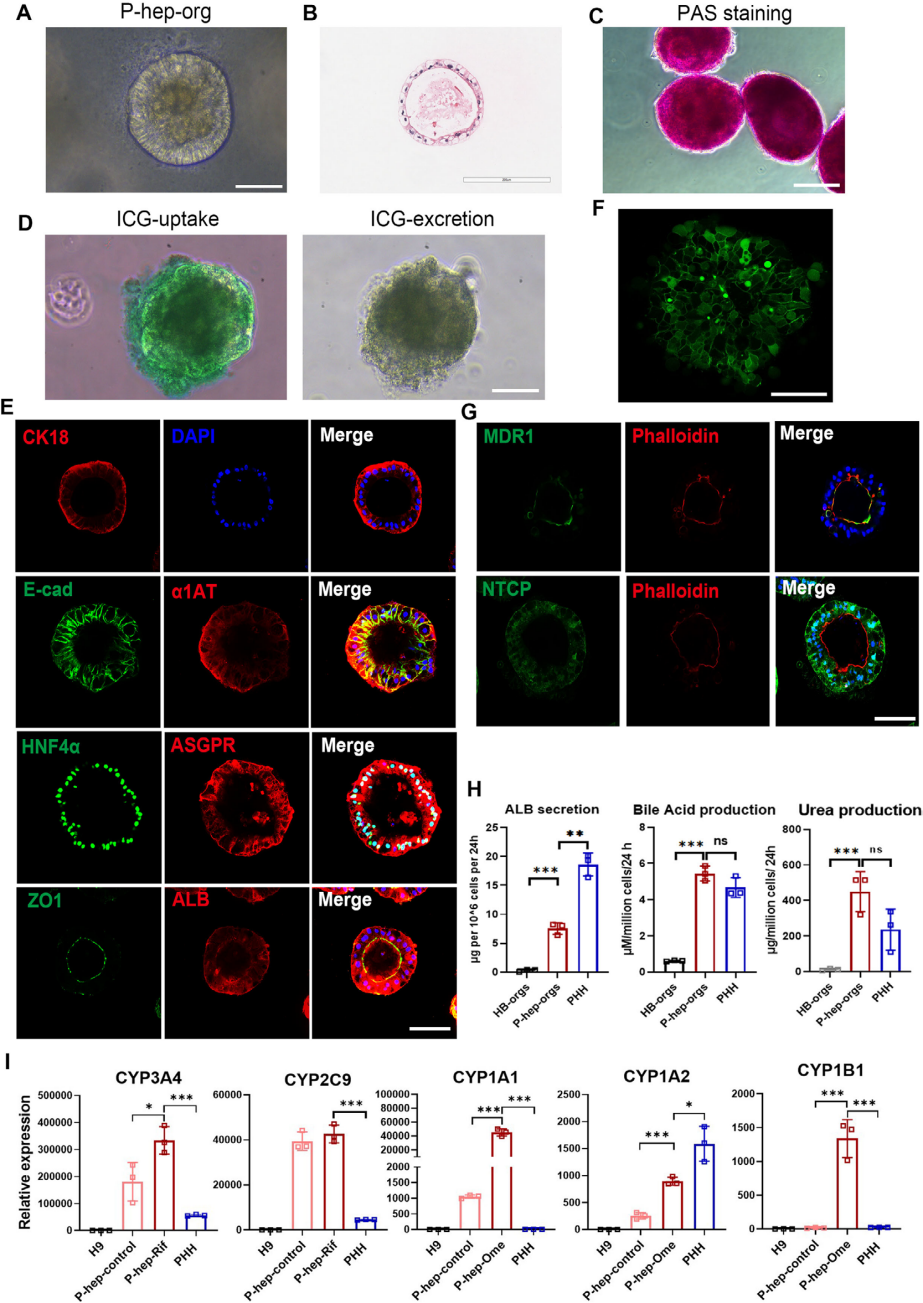
Characterisation of HB‐orgs–derived mature P‐hep‐orgs. (A) Representative morphology of P‐hep‐orgs. Scale bar = 100 μm. (B) H&E staining for P‐hep‐orgs. Scale bar = 200 μm. (C) PAS staining of P‐hep‐orgs. Scale bar = 100 μm. (D) ICG uptake and excretion assays of P‐hep‐orgs. Scale bar = 100 μm. (E) Immunostaining for mature hepatocyte markers in P‐hep‐orgs. Nuclei were stained with DAPI. Scale bar = 100 μm. (F) Bile canaliculi staining using CDFDA. Scale bar = 50 μm. (G) Immunostaining for polarised markers in P‐hep‐orgs. Nuclei were stained with DAPI. Scale bar = 100 μm. (H) Functional characterisation for the secretion of albumin, bile acid and urea. (I) The relative expression levels of CYP family genes were analysed by qRT‐PCR among different cells. Results were presented as mean ± SD. *p < 0.05, **p < 0.01 and ***p < 0.001.

Hepatocyte polarisation is essential for maintaining bile synthesis, secretion, and the exchange of components with sinusoidal blood [35]. Moreover, hepatocyte polarisation is influenced by cell–ECM interactions, as seen in sandwich and transwell models [36]. Therefore, we cultured HB‐orgs in HCM with 5% Matrigel, eventually producing polarised hepatocyte organoids (P‐hep‐orgs). P‐hep‐orgs displayed a typical columnar epithelial morphology (Figure 2A,B), exhibited functions of the storage of glycogen (Figure 2C) and the uptake and release of indocyanine green (Figure 2D). They also highly expressed mature hepatocyte markers, including HNF4α, ASGPR, α1AT, ALB and CK18 (Figure 2E). Notably, the well‐organised bile canaliculi‐like structures, clearly identified by carboxy‐dichlorofluorescein diacetate (CDFDA) staining (Figure 2F), and the apical marker MDR1 was co‐localised with F‐actin (Phalloidin), and the basal marker NTCP, were correctly positioned, along with ZO1 at tight junctions (Figure 2E,G), demonstrating that our P‐hep‐orgs were polarised with mature hepatocytes. Functional assessments revealed that P‐hep‐orgs exhibited a high capacity for secreting ALB and bile acid, moreover, 24 h after the treatment with 10 mM NH_4_Cl, P‐hep‐orgs produced a comparable level of urea to PHHs (Figure 2H), highlighting their strong ammonia elimination capacity. Furthermore, the response to the inducers is unique characteristic of mature hepatocytes, the expression of CYP3A4, CYP1A1, CYP1A2 and CYP1B1 in P‐hep‐orgs was significantly increased after the treatment with the inducers rifampicin (Rif) and omeprazole (Ome), and the expression levels of these CYPs and CYP2C9 were comparable or higher than those in PHHs with or without the inducers (Figure 2I). These results demonstrated that P‐hep‐orgs showed a higher level of maturity and had the potential to serve as a promising cellular source for the treatment of ALF and conditions related to ammonia toxicity, such as hepatoencephalopathy.

### 2.3 Single‐Cell Transcriptomic Analysis of P‐Hep‐Orgs

**FIGURE 3:**
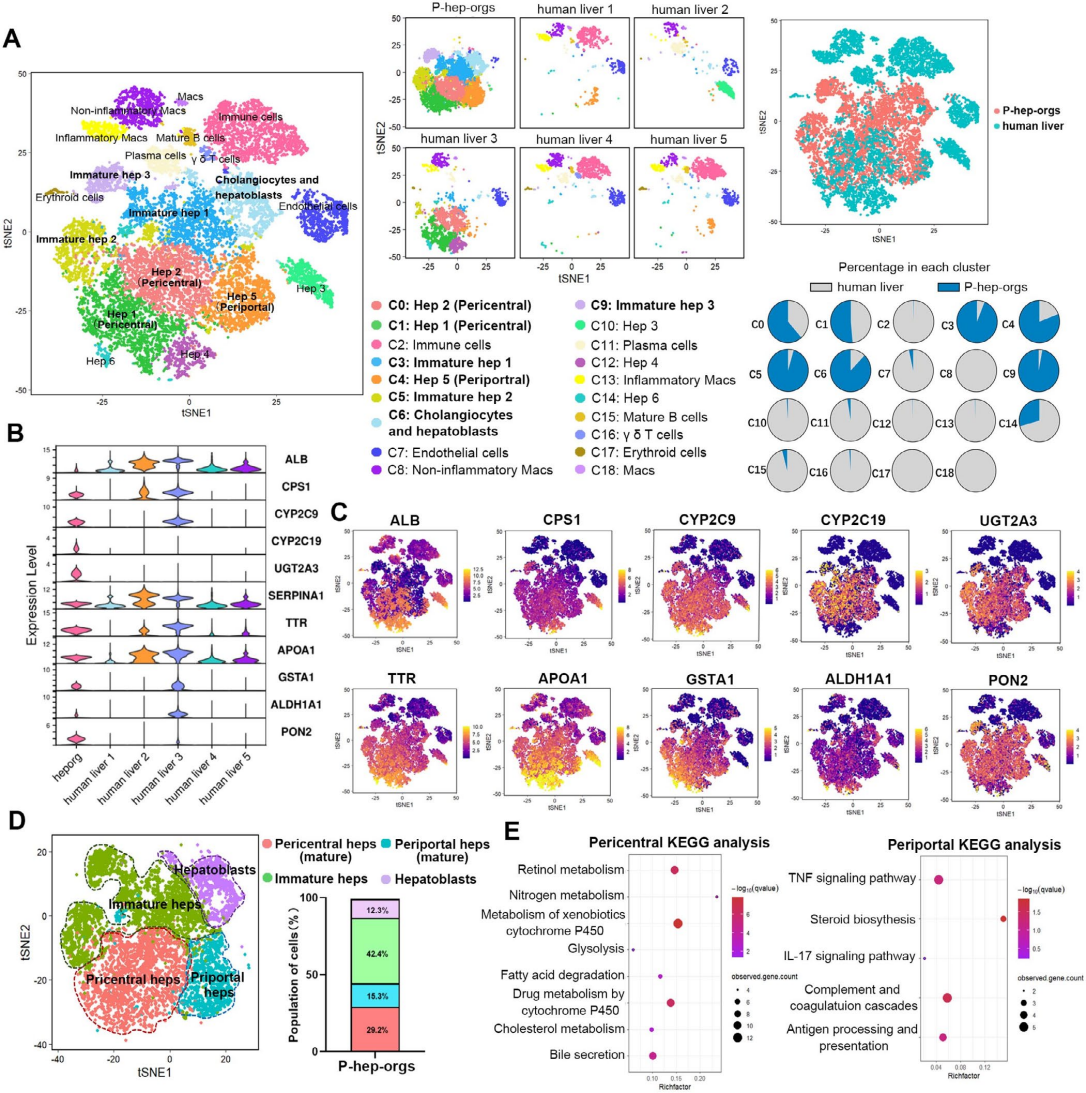
Single‐cell analysis of mature P‐hep‐orgs. (A) tSNE clustering of integrated single‐cell transcriptomes from P‐hep‐orgs (*n* = 7994) and primary human liver cells (*n* = 8443), coloured by cell type (left/middle) or original source (right). (B) Violin plot of mature hepatocyte genes in P‐hep‐orgs and primary human liver cells. (C) t‐SNE representations of hepatocyte functional genes in integrated single‐cell transcriptomes of P‐hep‐orgs and primary human liver cells. The colour scale indicated scaled gene expression levels. (D) t‐SNE clustering of single‐cell transcriptomes from P‐hep‐orgs coloured by cell types with the percentages of all cell types of P‐hep‐orgs in each cluster. (E) KEGG analysis comparing pericentral and periportal hepatocytes in P‐hep‐orgs.

scRNA‐seq of P‐hep‐orgs (*n* = 7994) at Day 8 was compared with primary human liver cells (*n* = 8443) [37]. Integrated analysis revealed that P‐hep‐orgs closely resembled mature primary hepatocytes originating from human liver 3 (Figure 3A), and expressed key mature hepatocyte genes (Figure 3B,C). Depending on the clusters of human livers, P‐hep‐orgs were able to be clustered into Hep 1, 2, 5, immature Hep 1, 2, 3, cholangiocytes and HBs (Figure 3A), and could be further classified as pericentral and periportal hepatocytes, immature hepatocytes and HBs based on gene expression signature and correlation analyses (Figures 3D and S6A,B). Pericentral hepatocytes are involved in drug metabolism and glycolysis, while periportal hepatocytes function in complement activation, steroid biosynthesis and immune stimulation [38], which were consistent with our KEGG analyses (Figure 3E). Drug metabolism–related genes (GSTA2, CYP2C9, OAT, UGT2A3) and urea cycle genes (CPS1, SAA1, CP) were enriched in pericentral and periportal hepatocytes of P‐hep‐orgs, respectively (Figure S6C). HB‐specific genes (KRT8, SOX9, KRT19) were enriched in HB cluster (Figure S6C,D), demonstrating that our definition of P‐hep‐org clusters was appropriate. Therefore, approximately 43% of P‐hep‐orgs were defined as mature hepatocytes (29% pericentral hepatocytes, 15% periportal hepatocytes), which closely resembled PHHs at single‐cell transcriptome level (Figure 3D).

To further compare P‐hep‐orgs with PHHs at single‐cell transcriptome level, we analysed differentially expressed genes in mature hepatocytes from Hep 1–5 clusters of both groups. GO enrichment revealed that mature PHHs highly expressed genes involved in immune response, complement activation, fatty acid metabolism and P450 pathway, while mature P‐hep‐orgs showed higher expression of biosynthetic process genes (Figure S7A). Although PHHs had superior metabolic abilities, specific genes such as CYP2C19, UGT2A3, ABCB1 and ABCC2 were more highly expressed in mature P‐hep‐orgs, demonstrating the comparable metabolic capacity between mature P‐hep‐orgs and PHHs (Figure S7B).

### 2.4 A Scalable 3D Suspension System for HB‐Org Expansion and the Generation of P‐Hep‐Orgs in Bioreactors

**FIGURE 4:**
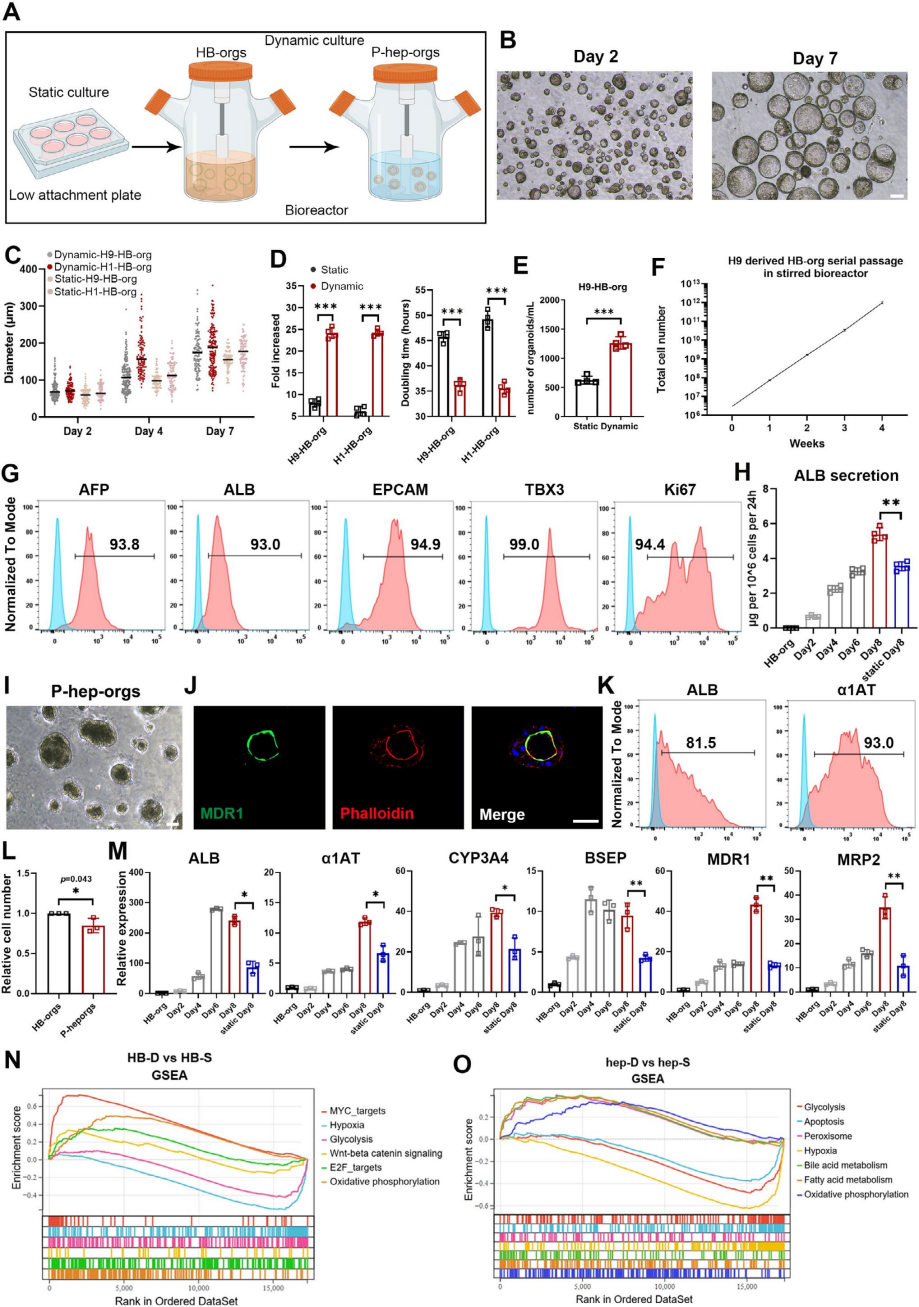
Large‐scale generation of HB‐orgs and P‐hep‐orgs in bioreactors. (A) Schematic illustration of scalable expansion culture for HB‐orgs and P‐hep‐orgs under 3D dynamic suspension in bioreactors. (B) Representative morphologies of HB‐orgs in bioreactors. Scale bar = 100 μm. (C) Average diameters of HB‐orgs at different time points under static and dynamic culture conditions. (D, E) Fold increments, doubling times (D) and average number of organoids (E) for HB‐orgs in static/dynamic culture systems. (F) Cumulative cell numbers of H9 cell derived HB‐orgs across four serial passages within 4 weeks in bioreactors. (G) The percentages of hepatoblast markers in dynamically cultured HB‐orgs. (H) The dynamics of ALB secretion in bioreactors compared with those in static conditions. (I) Representative morphologies of P‐hep‐orgs in bioreactors on Day 8. Scale bar = 100 μm. (J) Immunostaining for polarity markers MDR1 and F‐actin (Phalloidin) in P‐hep‐orgs in bioreactors. Nuclei were stained with DAPI. Scale bar = 100 μm. (K) Expression of mature hepatocyte markers in P‐hep‐orgs cultured in bioreactors. (L) Relative cell number of P‐hep‐orgs derived from expanded HB‐orgs in bioreactors from three independent cultures. (M) Dynamic expression of mature hepatocyte genes in P‐hep‐orgs in bioreactors analysed by qRT‐PCR and compared with those in static suspension condition. (N, O) GSEA analysis showing enriched pathways in HB‐D (N) and Hep‐D (O). Results were presented as mean ± SD. *p < 0.05, **p < 0.01 and ***p < 0.001.

To scale up HB‐orgs and P‐hep‐orgs, we developed a spinner flask‐based 3D suspension culture system (Figure 4A). This dynamic culture improves the aeration and nutrient distribution, increasing cell yield [27]. In bioreactors, HB‐orgs grew significantly larger (Figure 4B), reaching an average diameter of 180 μm at Day 7 (Figure 4C), and the proliferation ability was notably higher than those in static system, with a 24‐fold cell increment and the reduction of doubling time to 36 h (Figure 4D). By Day 7, over one thousand organoids per millilitre were generated under dynamic conditions (Figure 4E). Although the expansion ability was significantly augmented under dynamic culture condition, the heterogeneity in the diameter of HB‐orgs also was increased compared with those cultured under static culture condition (Figure 4C), indicating that this culture system needs to be optimised in the future. Furthermore, we assessed the long‐term culture capacity of H9 cell‐derived HB‐orgs in spinner flasks. HB‐orgs could be serially passaged and the cell number could be reached to a theoretical total 10^12^ after the expansion from 3 million cells within 4 weeks (Figure 4F). The expanded HB‐orgs still highly expressed HB and proliferation markers (Figure 4G).

We also differentiated large‐scale P‐hep‐orgs from expanded HB‐orgs in the same dynamic system. Eight days after the differentiation in bioreactors, the levels of ALB secretion gradually were increased and exceeded to those in the concurrent batch of P‐hep‐orgs which were differentiated in static culture condition (Figure 4H). Immunostaining of MDR1 and F‐actin confirmed that the polarisation was also induced and maintained under dynamic system (Figure 4I,J), and mature hepatocyte markers ALB and α1AT remained highly expressed (Figure 4K). In addition, P‐hep‐orgs retained similar cell numbers to those of the expanded HB‐orgs after the differentiation in large scale using bioreactors (Figure 4L) and expressed higher levels of maturation‐related genes such as ALB, α1AT, MDR1 and MRP2 (Figure 4M), indicating that the dynamic system better supported the culture and maintenance of mature hepatocytes in vitro.

To further explore gene expression in organoids under dynamic culture, we performed RNA‐seq analysis and compared organoids cultured statically (HB‐S, hep‐S) and dynamically (HB‐D, hep‐D). In HB‐D, 1274 genes were upregulated, with GO and KEGG analysis showing enrichment in cell proliferation, cell cycle and telomere maintenance pathways (Figure S8A–C). Similarly, 2501 genes were upregulated in hep‐D, highlighting liver‐specific functions like drug metabolism, cytochrome P450 activity, bile secretion and steroid/retinol metabolism (Figure S9A–C). Gene set enrichment analysis showed hypoxia‐ and glycolysis‐related genes enriched in statically cultured organoids, while oxidative phosphorylation‐related genes were more prominent in dynamic cultures (Figure 4N,O). These results indicated that dynamic culture improved oxygen and nutrient availability, enhancing energy metabolism and promoting the proliferation of HB‐orgs and maturation of P‐hep‐orgs.

These results confirmed that our scalable system had the potential to produce sufficient hepatocytes to meet global needs for preclinical and clinical cell‐based therapies, toxicology studies and drug screening and development.

### 2.5 Differentiation of HB‐Orgs Into Cholangiocyte‐Like Cell Organoids

**FIGURE 5:**
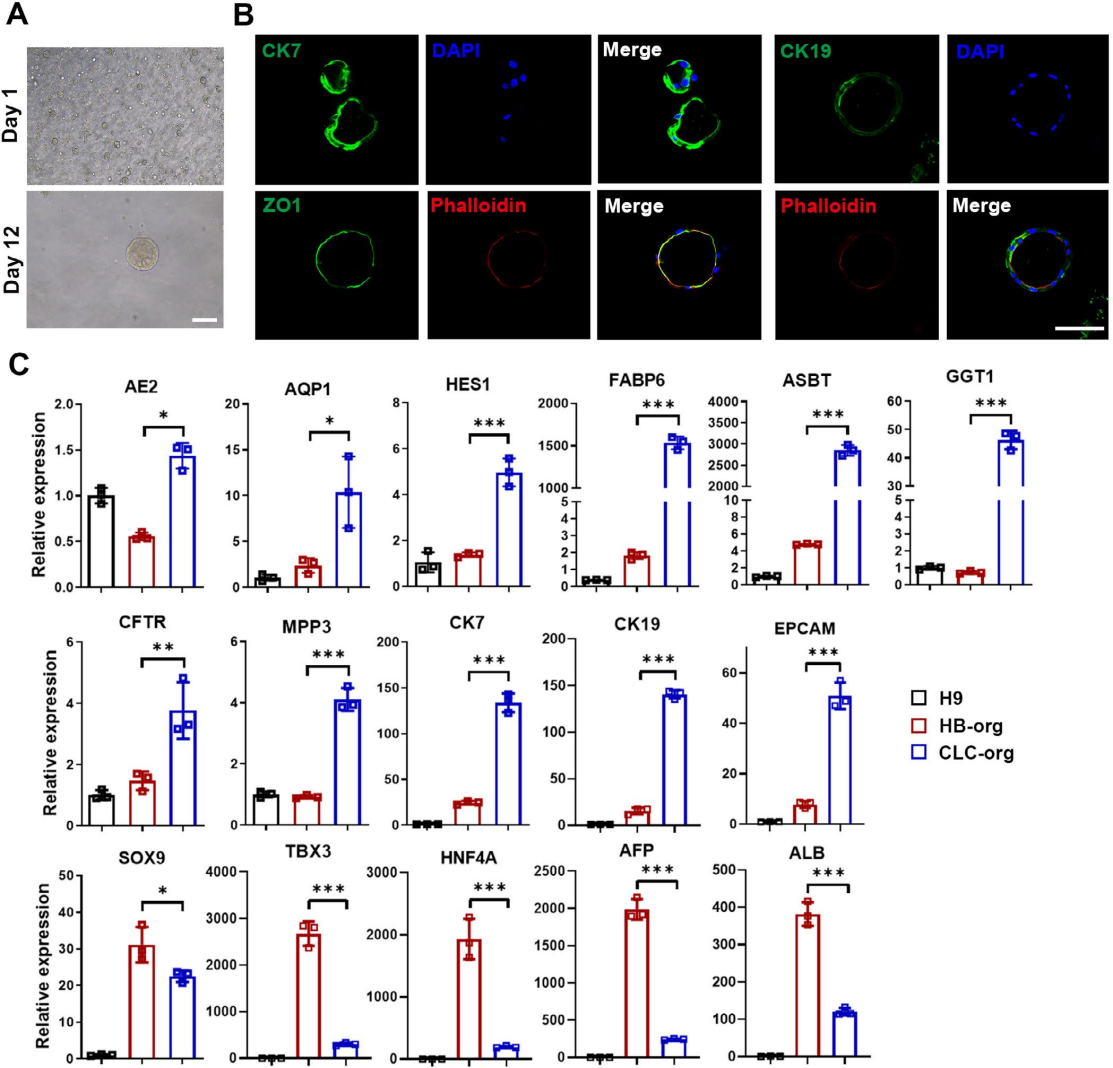
The differentiation of HB‐orgs towards CLC‐orgs. (A) Representative morphology images of CLC‐orgs. Scale bar = 100 μm. (B) Immunostaining for cholangiocyte markers in CLC‐orgs. Nuclei were stained with DAPI (blue). Scale bar = 100 μm. (C) The relative expression levels of cholangiocyte and hepatocyte‐related genes were analysed by qRT‐PCR among H9 cells, HB‐orgs and CLC‐orgs (*n* = 3 for each group). Results were presented as mean ± SD. *p < 0.05, **p < 0.01 and ***p < 0.001.

To confirm the bipotency of HB‐orgs, they were dissociated into single cells and differentiated into cholangiocyte‐like cells (CLC) under 3D suspension culture. Twelve days after the differentiation, CLC organoids (CLC‐orgs) were formed (Figure 5A) and expressed biliary markers CK7 and CK19, with co‐expressing ZO1 and F‐actin localised at their inner side, indicating a polarised epithelial cell structure (Figure 5B). Cholangiocyte‐related genes were upregulated and the expression of hepatocyte genes AFP and ALB was significantly decreased (Figure 5C). These results demonstrated that HB‐orgs were bipotent and could differentiate into both polarised P‐hep‐orgs and CLC‐orgs under our 3D suspension culture system.

### 2.6 Modelling of Hepatic Lipid Accumulation Using P‐Hep‐Orgs

**FIGURE 6:**
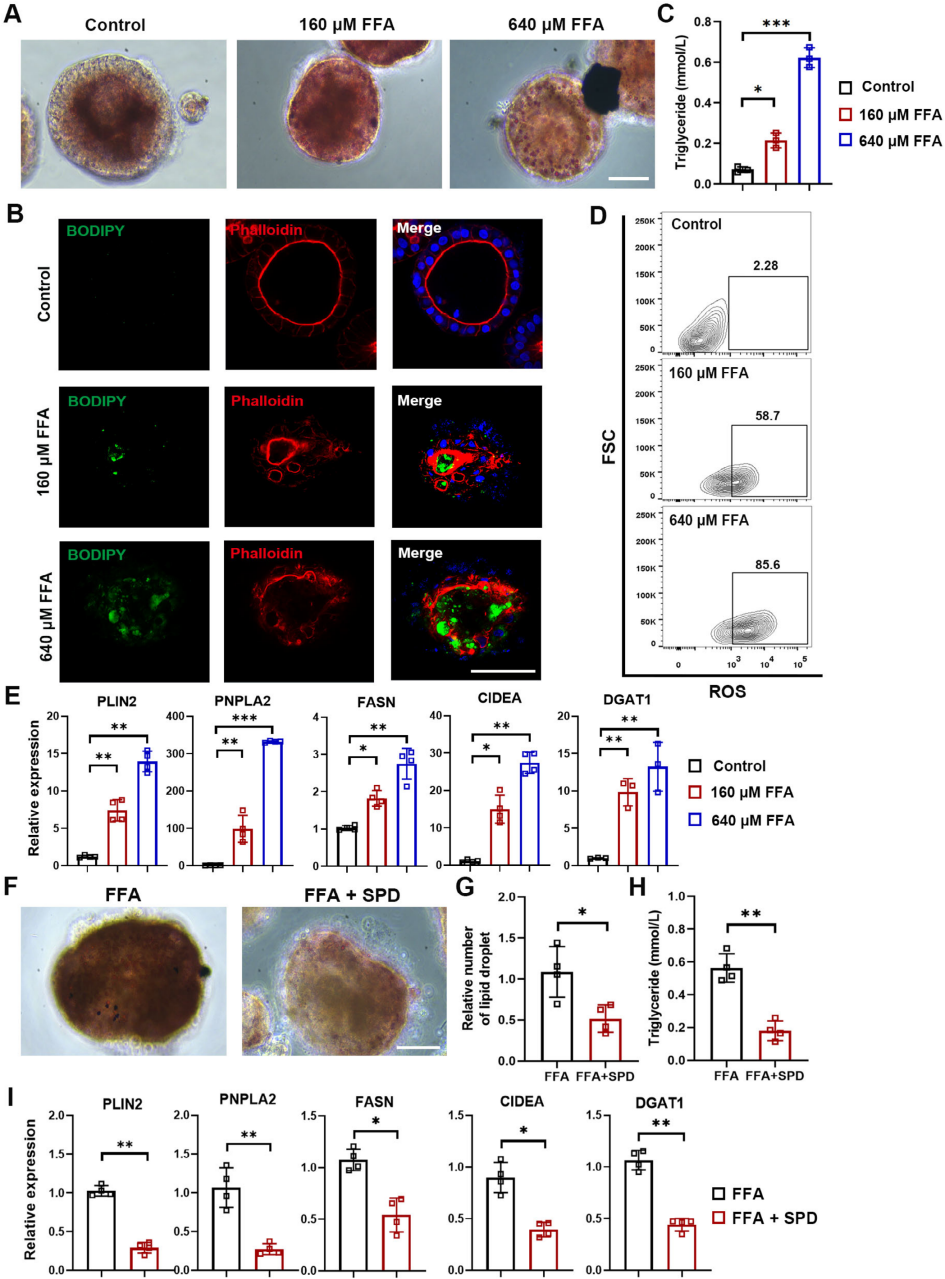
Disease modelling for hepatic lipid accumulation using P‐hep‐orgs. (A) Representative images of lipid droplets formation in P‐hep‐orgs treated with different FFA concentrations, stained by Oil Red O (scale bar = 50 μm). (B) Co‐immunostaining for lipid droplets (BODIPY) and F‐actin (Phalloidin) in FFA‐treated P‐hep‐orgs. Nuclei were stained with DAPI. Scale bar = 100 μm. (C) Quantitation of triglyceride levels in P‐hep‐orgs. (D) ROS levels were assessed by DCFH‐DA labeling. (E) qRT‐PCR analysis of lipid metabolism‐related genes. (F) Representative images showing lipid droplet reduction in P‐hep‐orgs after SPD treatment. Scale bar = 50 μm. (G, H) Quantitation of lipid droplet (G) and secreted triglyceride concentration (H) after SPD treatment. (I) qRT‐PCR analysis of lipid metabolism‐related genes after SPD treatment. Results were presented as mean ± SD. *p < 0.05, **p < 0.01 and ***p < 0.001.

Hepatic steatosis is a consequence of lipid acquisition and accumulation of exceeding lipid disposal, and it can further result in nonalcoholic fatty liver disease [39]. Therefore, we sought to evaluate whether our P‐hep‐orgs could be employed to model the pathological changes of hepatic lipid accumulation. P‐hep‐orgs were treated with various concentrations of free fatty acid (FFA, oleic acid: palmitic acid = 2:1) for 48 h. Oil Red O and BODIPY staining revealed significant lipid accumulation (Figure 6A,B), with disrupting the polarised structure of the organoids, as indicated by F‐actin staining (Figure 6B). Triglyceride levels in the supernatants were increased significantly in FFA‐exposed organoids (Figure 6C). Flow cytometry analysis also showed elevated reactive oxygen species (ROS) production in FFA‐treated organoids (Figure 6D), a marker of nonalcoholic steatohepatitis‐related hepatocyte damage [40]. Lipid accumulation‐related genes such as FASN, CIDEA, DGAT1, PLIN2 and PNPLA2 were upregulated in P‐hep‐orgs following FFA treatment (Figure 6E). These results highlighted the expression changes associated with hepatic lipid accumulation in our model.

To assess the effects of therapeutic compounds, we applied spermidine (SPD), a natural polyamine known to protect against diet‐induced obesity in animal models [41]. The treatment with SPD significantly reduced both lipid droplet numbers and triglyceride levels (Figure 6F–H), alongside the decrease on the expression of lipid‐associated genes (Figure 6I). These results demonstrated that P‐hep‐orgs provided a reliable model for studying hepatic steatosis and its treatment.

### 2.7 P‐Hep‐Orgs Exhibited the In Vivo Function by Improving the Survival of Mice With ALF

**FIGURE 7:**
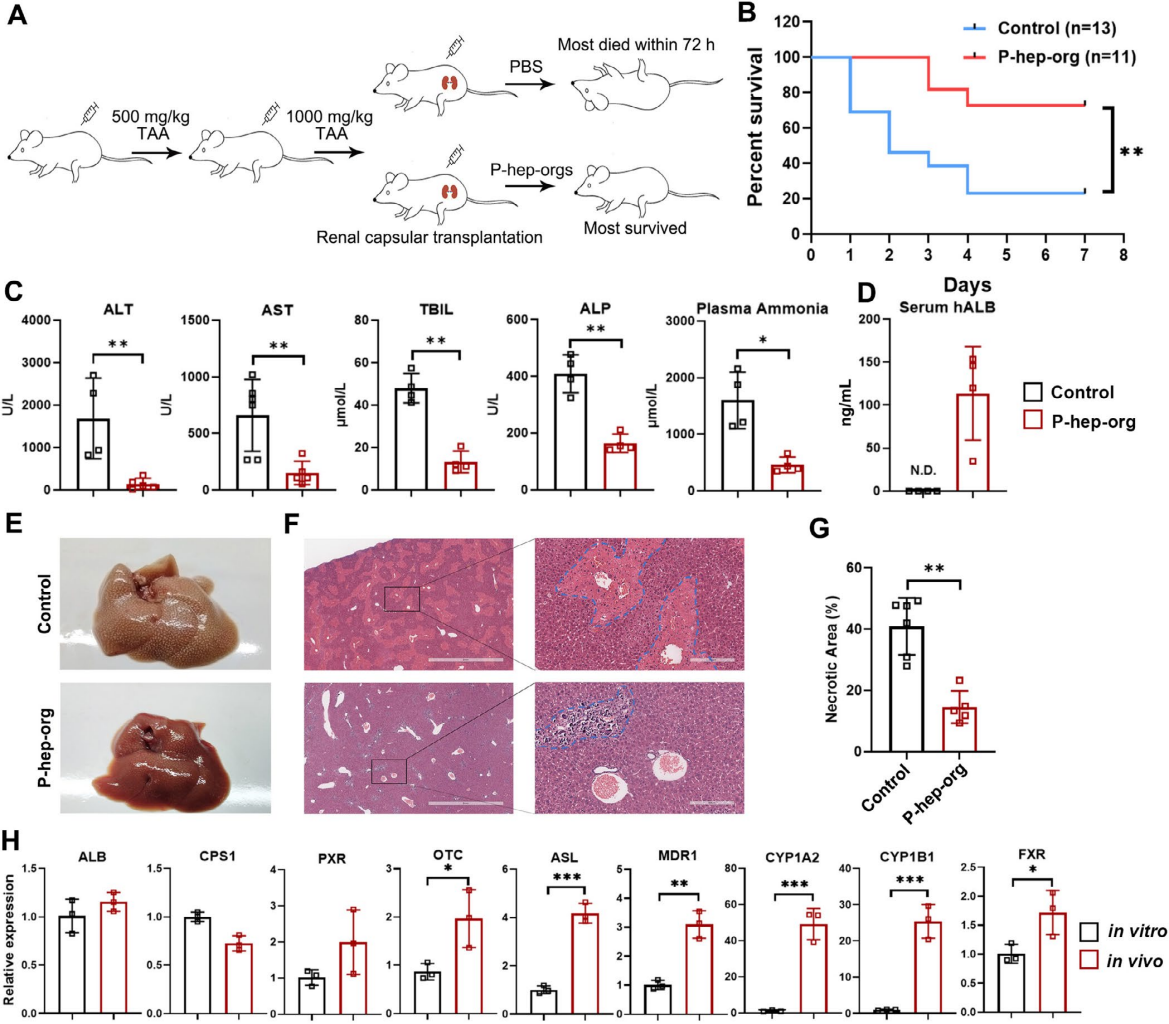
P‐hep‐orgs rescue of acute liver failure. (A) Schematic illustration of the generation of ALF mice and rescue via the transplantation of P‐hep‐orgs. (B) Kaplan–Meier survival curves of ALF mice with or without the transplantation of P‐hep‐orgs. (C) Levels of ALT, AST, TBIL, ALP and plasma ammonia at Day 3 in PBS (control) and P‐hep‐org–transplanted mice. (D) Human‐specific albumin levels at Day 7 after the transplantation of P‐hep‐orgs. (E, F) Images of the livers (E) and H&E staining at Day 7 the transplantation of P‐hep‐orgs. Scale bar = 2 mm and 200 μm. (G) Statistical analysis of liver necrosis from H&E‐staining of control mice and P‐hep‐org–transplanted mice. (H) qRT‐PCR analysis of mature hepatocyte–related genes in vitro P‐hep‐orgs and in vivo engrafted P‐hep‐orgs. Results were presented as mean ± SD. *p < 0.05, **p < 0.01 and ***p < 0.001; N.D. = not detectable.

To evaluate the function of P‐hep‐orgs in vivo, we induced ALF in mice via intraperitoneal injection of thioacetamide (TAA) (Figure 7A). Following TAA treatment, mice exhibited typical symptoms of ALF, including elevated serum ALT, AST, ALP, total bilirubin (TBil) and plasma ammonia levels, with 10 of 13 mice dying within 7 days without rescue treatment (Figure 7B). Remarkably, the transplantation of P‐hep‐orgs into the renal subcapsular space protected 8 of 11 mice from ALF (Figure 7B) with significantly reduced serum ALT, AST, TBil and plasma ammonia levels 3 days post‐treatment compared with untreated TAA mice (Figure 7C). H&E staining revealed fewer necrotic regions in transplanted mice (Figure 7E–G), confirming the recovery of mouse livers. Human‐specific ALB protein was detected in mouse serum (Figure 7D), and the expression levels of hepatocyte functional genes in grafted P‐hep‐orgs were comparable or higher than those in vitro (Figure 7H), indicating that transplanted P‐hep‐orgs survived, functioned and supported liver regeneration in ALF mice.

## 3 Discussion

The shortage of functional hepatocytes for cell‐based therapy and drug development as well as disease modelling highlights the urgent need for the establishment of a scalable expansion system for hepatocytes in vitro [42]. Although previous studies reported that HBs or hepatocyte organoids derived from hPSCs or PHH could be expanded in vitro to 10^8^–10^10^ accumulatively in cell number [27, 43, 44], it was still a challenge to achieve a large number of cells with a more convenient and economical way [45, 46], and those hepatocytes did not exhibit the authentic state of hepatocytes in the liver [10]. In this study, we presented an efficient method for generating mature P‐hep‐orgs from hESC‐derived expandable HB‐orgs using 3D dynamic suspension culture in bioreactors. By developing a defined culture condition to expand HB‐orgs through modulating signalling transduction with growth factors and small molecules, we found that only 5% Matrigel was quite appropriate for supporting the continuous growth of HB‐orgs, making our system have the capacity of suitability for further scale‐up. Furthermore, as compared with previous studies [14, 47], our HB‐expansion medium excluded some high‐concentration growth factors including R‐spondin, Wnt3a, HGF, FGF7 and FGF10, making our system more economical. Moreover, the polarised and mature P‐hep‐orgs could be further induced directly from HB‐orgs. P‐hep‐orgs exhibited key liver characteristics, including hepatic columnar polarisation, well‐defined apical and basolateral membranes, tight junctions and bile canaliculi. The scRNA‐seq analysis confirmed that hESC‐derived HB‐orgs resembled human fetal liver at 6 weeks [33, 38], and 43% of P‐hep‐orgs were comparable to PHHs [37], demonstrating that our differentiated HB‐orgs and P‐hep‐orgs were very similar to primary human fetal and adult hepatocytes.

By utilising spinner bioreactors to enhance oxygen and nutrient distribution, HB‐orgs can achieve a cumulative cell count of up to 10^12^ within just 4 weeks under 3D suspension dynamic culture conditions, starting from an initial population of 3 million cells. Furthermore, spinner bioreactors support the differentiation of mature P‐hep‐orgs from HB‐orgs, significantly enhancing their maturity compared with organoids cultured in static conditions. The development of expandable hESC‐derived HB‐orgs from a minimal initial number of hESCs marks a transformative breakthrough in our system. This innovation eliminates the reliance on producing large quantities of hESCs, a process traditionally associated with prohibitive costs, for scaling up hepatocyte generation. Instead, our approach enables the rapid and cost‐effective expansion of HB‐orgs, which can be differentiated into an equivalent number of mature hepatocytes in just 1 week. This system offers a convenient and efficient solution to overcome the challenges of rapidly producing mature hepatocytes or hepatocyte organoids, potentially meeting global demands across various applications in research, drug development and regenerative medicine. Although our approach successfully generated an optimal number of functional hepatocytes for various applications, further advancements are needed to enhance the stability of large‐scale bioreactors. For instant, using novel bioreactor systems, including pulsed electromagnetic‐wave motion–based system [48], perfusion system [49] and scaffold‐supported system [50, 51, 52] to improve the homogeneity and inter‐batch stability of organoids. In addition, incorporating artificially synthesised, highly bioactive extracellular matrices into the large‐scale production process is essential [53, 54]. This step would improve the safety of clinical‐grade functional hepatocytes while reducing production costs, paving the way for broader clinical applications [55, 56].

In conclusion, our approach successfully overcame technical difficulty in large‐scale production of mature and polarised hepatocyte organoids by generating expandable, storable HB‐orgs from hESCs under 3D dynamic suspension conditions. This provides a valuable cell source for cellular therapies, disease modelling and drug development. In addition, as polarised organoids, they offer an ideal platform for studying the mechanisms of liver polarisation and depolarization, particularly in relation to hepatitis virus infection, where the polarisation is critical for viral infection and particle secretion [36].

## 4 Materials and Methods

Materials and methods are provided in Supplementary methods.

## Author Contributions

Y.Y.D. and H.B.W. conceived and designed the experiments, H.B.W. performed the experiments, collected and analysed data, prepared the manuscript. S.P.L. involved in the animal experiments. J.H.X. and X.Y.Z. involved in bioinformatics analysis. J.W., Y.Y.W., X.L.T., S.C., J.W., H.H.S., W.P.Z., C.X.C. and Y.J.F. contributed to the collection of the data. L.X.L. provided materials. Y.Y.D. and J.W. provided financial support.

## Ethics Statement

All animal experiments were performed according to our experimental protocols approved for the Use and Care of Laboratory Animals by the Animal Ethics Committee of School of Medicine of South China University of Technology (approval number: 2019073).

## Conflicts of Interest

Yuyou Duan and Haibin Wu are inventors on a patent application owned by South China University of Technology. The other authors declare no conflicts of interest.

## Supporting information


**Data S1.** Supporting Information.

## Data Availability

The data that support the findings of this study are available from the corresponding author upon reasonable request. The RNA‐seq and scRNA‐seq datasets have been deposited in Gene Expression Omnibus (GEO) with accession number GSE240097 and GSE265845.
