# Supplementary material for: Large‐Scale Production of Expandable Hepatoblast Organoids and Polarised Hepatocyte Organoids From hESCs Under 3D Static and Dynamic Suspension Conditions
Source: Cell Prolif. 2025 Feb 8;58(7):e70001. doi: 10.1111/cpr.70001 (PMC12240639; doi:10.1111/cpr.70001)
Supplement: Supplementary file 1 — Data S1. Supporting Information. [file CPR-58-e70001-s001.docx]

Supporting Information

**Large-scale Production of Expandable Hepatoblast Organoids and Polarized Hepatocyte Organoids From hESCs Under 3D Static and Dynamic Suspension Conditions**

Haibin Wu^1,2†^, Jue Wang^2†^, Shoupei Liu^1,2^, Yiyu Wang^1,2^, Xianglian Tang^2^, Jinghe Xie^2,3^, Ning Wang^1,2^, Huanhuan Shan^1,2^ Sen Chen^1,2^, Xueyan Zhang^1,2^, Weiping Zeng^2^, Chuxin Chen^1,2^, Yinjie Fu^1,2^, Liangxue Lai^4^, Yuyou Duan^1,2,5,6^*

**Affiliations:**

^1^Laboratory of Stem Cells and Translational Medicine, Institute for Medical Research, the Second Affiliated Hospital, School of Medicine, South China University of Technology; Guangzhou, 510006, China.

^2^Laboratory of Stem Cells and Translational Medicine, Institute for Life Science, School of Medicine, South China University of Technology; Guangzhou, 510006, China.

^3^School of Biomedical Sciences and Engineering, South China University of Technology, Guangzhou International Campus; Guangzhou, 511442, China.

^4^Key Laboratory of Regenerative Biology, South China Institute for Stem Cell, Biology and Regenerative Medicine, Guangzhou Institutes of Biomedicine and Health, Chinese Academy of Sciences; Guangzhou, 510700, China.

^5^National Engineering Research Center for Tissue Restoration and Reconstruction, South China University of Technology; Guangzhou, 510006, China.

^6^The Innovation Centre of Ministry of Education for Development and Diseases, the Second Affiliated Hospital, School of Medicine, South China University of Technology; Guangzhou 510006, China.

*Corresponding author. Email: yuyouduan@scut.edu.cn

†These authors contributed equally to this work.

**Supplementary methods**

**Cell culture**

The hESC lines, H9 and H1 cells were obtained from WiCell Research Institute (Madison, WI, USA) under Materials Transfer Agreements (No. 19-W0512, 24-W0162, 24-W0163). H9 and H1 cells were stably cultured on mouse embryonic fibroblast feeder layers in DMEM/F12 medium (Gibco, C11330500BT) containing 20% knockout serum replacement (KSR, Gibco, 10828028), 1% non-essential amino acids (Gibco, 11140050), 0.1 mM 2-mercaptoethanol (Sigma, M3148), 1% GlutaMax I (Gibco, 35050061) and 10 ng/mL bFGF (PeproTech, 100-18B), and maintained in a humidified incubator at 37 °C and 5% CO_2_. The culture medium was refreshed daily, and hESC colonies were dissociated and split into small clumps by collagenase IV (Gibco, C0130) and then transferred onto a fresh feeder layers every 5-6 days. The hiPSC line (UH10 cells) was provided by Dr Liangxue Lai, cultured in mTeSR1 medium (Stem Cell Technologies, 85850) on Matrigel (Corning, 354277) coated plates, and maintained in a humidified incubator at 37 °C and 5% CO_2_. The culture medium was refreshed daily, and hiPSC colonies were dissociated and split into small clumps by ReLeSR (Stem Cell Technologies, 05872) and then transferred onto a freshly coated plates every 5-6 days.

**Differentiation of HB-orgs from hPSCs under 3D suspension culture condition**

To obtain hPSC aggregates in 3D suspension culture condition, hPSC colonies were dissociated into single cells by Gentle Cell Dissociation Reagent (Stem Cell Technologies, 100-0485), seeded in ultra-low attachment 6-well plates (Corning, 3471) at a cell density of 2 × 10^5^ cells/mL, and cultured in mTeSR1 medium with 10 μM Y27632 (MedChemExpress, HY-10071) for 1 day, maintained in a humidified incubator at 37 °C and 5% CO_2_. The medium was changed daily, and single cells from hPSCs formed aggregates spontaneously. This stage lasted for three days to allow the aggregated hPSC to proliferate. And then, for the generation of definitive endoderm (DE) spheres[1], hPSC aggregates were cultured in RPMI 1640 medium (Gibco, C11875500BT) supplemented with 100 ng/mL Activin A (Peprotech, 120-14) and 3 μM CHIR99021 (MedChemExpress, HY-10182) for one day, and transferred to RPMI 1640 medium supplemented with 100 ng/mL Activin A and 0.8% Knockout serum replacement (KSR, Gibco, 10828028) for the second day, then transferred to RPMI 1640 medium supplemented with 100 ng/mL Activin A and 8% KSR for the third day. To the differentiation of HB spheres, DE spheres were transferred to HDM medium that consisted of IMDM medium (Gibco, C12440500BT), 20% fetal bovine serum (ExCell Bio, FND500), 1% GlutaMax I, 0.3 mM 1-thioglycerol (Sigma, M6145), 100 nM dexamethasone (Sigma, D4902), 0.5% DMSO (MP Biomedical, 196055), 0.126 U/mL human insulin (Sigma, 91077C), 20 ng/mL FGF4 (Peprotech, 100-31), 20 ng/mL HGF (Peprotech, 100-39), 10 ng/mL BMP2 (Peprotech, 120-02) and 10 ng/mL BMP4 (Peprotech, 120-05), cultured for 6 days and the medium was changed daily.

In order to generate expandable HB-orgs, differentiated HB spheres were first dissociated into single cells with TrypLE (Gibco, 12604021), then single cells were seeded in ultra-low attachment 6-well plates at a cell density of 2 × 10^5^ cells/mL and cultured in HB-expansion medium that consisted of IMDM medium, 10% FBS, 1% ITSE (Neobioscience, 00-101-10), 1% NEAA, 1% GlutaMax I, 0.3 mM 1-thioglycerol, 10 mM Nicotinamide (Sigma, N0636), 5 μM CHIR99021 (MedChemExpress, HY-10182), 10 μM SB431542 (MedChemExpress, HY-10431), 10 μM Forskolin (MedChemExpress, HY-15371), 20 ng/mL BMP4, 20 ng/mL FGF4, 20 ng/mL EGF (Peprotech, AF-100-15) and 10 μM Y27632 for one day and cells reaggregated to form spheres within 24 hours. Next day, the culture medium was supplemented with dispersing 5% growth factor reduced Matrigel (Corning, 354230) using 1000 mL tips, and the medium was changed daily for 5-6 days and the typical morphology of HB-orgs would emerge within 5 days. For a long-term passaging of the organoids, HB-orgs were dissociated into single cells with TrypLE and split at 1:4-6 every 5-6 days, and 5% ice-cold growth factor reduced Matrigel was added when seeding cells at each passage. For the cryopreservation, HB-orgs were dissociated into small fragments by gently pipetting cells up and down for ten times with TrypLE and frozen in mFreSR (Stem Cell Technologies, 05855). The frozen small fragments could be thawed following standard procedures and cultured to expand in the HB-expansion medium with 10 μM Y27632 for the first day.

**The differentiation of HB-orgs into P-hep-orgs**

HB-orgs were directly transferred to hepatocyte culture medium (HCM) supplemented with SingleQuots minus EGF, supplemented with 1% B27, 100 nM dexamethasone, 20 ng/mL FGF4, 20 ng/mL HGF and 40 ng/mL Oncostatin M, 5% ice-cold growth factor reduced Matrigel was added into the medium during HB-orgs were transferred. The medium was changed every other day for at least 6 days.

**Scalable generations of HB-orgs or P-hep-orgs in spinner bioreactors**

For large-scale production of HB-orgs, the organoids at passage 3 or 4 were first dissociated into single cells as mentioned above, then seeded in ultra-low attachment 10 cm dishes at a cell density of 1 × 10^5^ cells/mL, supplemented with 5% growth factor reduced Matrigel and cultured in HB-expansion medium. 24 hours later, single cells were aggregated into small organoids, then they were transferred to a disposable 125-mL stirred bioreactors (Corning, 3152) for dynamic culture with 3 million cells in 30 mL HB-expansion medium. The speed of rotation was set at 60 rpm all the way. Fresh medium was supplemented into the bioreactor every day until reached a total volume of 90 mL. To differentiate HB-orgs into P-hep-orgs in spinner bioreactors, HB-orgs were collected from the stirred bioreactor and transferred into ultra-low attachment 10 cm dishes, cultured with HCM supplemented with SingleQuots minus EGF, supplemented with 1% B27, 100 nM dexamethasone, 20 ng/mL FGF4, 20 ng/mL HGF, 40 ng/mL Oncostatin M, and 5% ice-cold growth factor reduced Matrigel. 24 hours later, all organoids were transferred into a new disposable 125-mL stirred bioreactors for dynamic culture with nearly 30 million cells in 30 mL complete HCM medium. The speed of rotation was set at 60 rpm all the way. Fresh medium was supplemented into the bioreactor every two days until reached a total volume of 60 mL. P-hep-orgs and their RNA as well as culture medium of P-hep-orgs were collected every 2 days for the analyses during the differentiation for 8 days.

**Differentiation of HB-orgs into** **cholangiocyte-like cell (CLC) organoids**

In order to obtain CLC-orgs, HB-orgs were dissociated into single cells with TrypLE and seeded in ultra-low attachment 24-well plates at a cell density of 1 × 10^5^ cells/mL and cultured in a mixture of 40% growth factor reduced Matrigel and 60% cholangiocyte differentiation medium containing DMEM/F12 medium, 1% B27, 1% GlutaMax I, 10 mM Nicotinamide supplemented with 50 ng/mL EGF, 25 ng/mL HGF and 5 ng/mL TGFβ1 (Peprotech, 100-21). The medium was changed every 3 days, and the cells were cultured for 12 days.

**Development of hepatic lipid accumulation model with P-hep-orgs**

P-hep-orgs were cultured in complete HCM medium supplemented with 160 or 640 μM FFA (oleic acid: palmitic acid = 2:1, Sigma, O1383, P0500) for up to 4 days. The control group were maintained in normal medium without FFA. 200 μM spermidine (SPD, Sigma, S0266) was added into the medium for 48 hours in order to relieve the phenotype of FFA-induced lipid accumulation.

**Quantitative reverse transcription polymerase chain reaction (qRT‑PCR)**

Total RNAs were extracted using RNAiso Plus kit (Takara, 9109) following the manufacturer’s instructions. 1 μg RNA was used to synthesize cDNA by using the PrimeScript RT master mix (Takara, RR036B). qRT-PCR was performed in triplicate using PowerUp SYBR Green (Thermo, A25742) on the Quant Studio 1 Real-Time PCR system (ABI, Thermo, USA). CT values for each sample were normalized to the corresponding expression of housekeeping gene glyceraldehyde-3-phosphate dehydrogenase (GAPDH). The relative gene expression levels were quantified using the 2^−ΔΔ CT^ method. The primer sequences for qRT-PCR used in the present study were listed in Supplementary Table. 1.

**Flow cytometry analysis**

Single cell suspensions were obtained by the dissociation with TrypLE for 5-10 minutes at 37 °C, then single cells were fixed and permeabilized by Transcription Factor Staining Buffer Set (Invirtrogen, 00-5523-00) according to the manufacturer’s manual. After blocking with goat (Boster Biological Technology, AR0009) or donkey serum (Servicebio, G1217), cells were stained with the specific antibodies listed in Table S2 and passed through 40 μm cell strainers. Finally, the stained cells were analyzed by FACS Celesta flow cytometer (BD, USA).

**Immunofluorescence (IF) staining**

The organoids were collected at specific time points, washed with PBS, and fixed with 4% paraformaldehyde overnight at 4 °C, then permeabilized with 0.5% Triton X‐100 for 20 minutes, and blocked with goat or donkey serum for 60 minutes. Three times washes with PBS were included between each step. Next the organoids were incubated with primary antibodies diluted in PBS at 4°C overnight, next day followed by the incubation in PBS containing secondary antibodies for 1 hour in the dark at room temperature. Nuclei were counterstained by the staining with DAPI (Beyotime Biotechnology, C1006) for 5 minutes. The immunostaining images were observed using the single photon confocal microscopy (Ti-E A1, Nikon, Japan). Antibodies used in this study were listed in Supplementary Table. 2.

**Western blot (WB) analysis**

Cells were lysed in ice-cold RIPA lysis buffer (Solarbio, R0020) supplemented with PMSF (Beyotime Biotechnology, ST505), protease inhibitor (Beyotime Biotechnology, P1005) and phosphatase inhibitor (Beyotime Biotechnology, P1081). Protein concentrations were determined using Bicinchoninic Acid Protein Assay Kit (Biosharp, BL521A) according to the manufacturer’s instructions. Western blots were performed in standard approach. Antibodies used in this study were listed in Supplementary Table. 2.

**Rescue of mice with acute liver failure (ALF) by the transplantation of P-hep-orgs into** **mouse renal capsules**

All animal procedures were approved by the Research Ethics Committee of School of Medicine at South China University of Technology (Ethical approval No. 2019073). Fah ^–/–^ /Rag2 ^–/–^ /Il2rg ^–/–^ (FRG) mice were a gift from Dr. Xin Wang[2] with a Material Transfer Agreement, and used to induce ALF. Mice were maintained with 7.5 mg/L NTBC in the drinking water. To induce ALF, mice (8-10 weeks) were administered with 500 mg/kg (body weight) thioacetamide (TAA) (Sigma, 163678) at day 1, and 1000 mg/kg TAA at day 2, through intraperitoneal injection. 18 hours after the second administration with TAA, PBS or 1.5 × 10^6^ cells of P-hep-orgs were injected into renal capsules directly. The blood samples were collected at 72 and 168 hours after the transplantation with cells or PBS, and all survival mice were sacrificed 168 hours after the transplantation for histological analysis.

**Serological detection of human albumin and mouse liver proteins**

The serum of mice was prepared by the centrifugation after mouse blood were collected from tail vein for detecting aspartate transaminase (AST, Njjcbio, C009-2) by Aspartate aminotransferase Assay Kit, alanine transaminase (ALT, Njjcbio, C010-2) by Alanine aminotransferase Assay Kit, alkaline phosphatase (ALP, Njjcbio, A059-2-2) by Alkaline phosphatase assay kit, total bilirubin (TBIL, Njjcbio, C019-1) by total bile acid (TBA, Njjcbio, E003-2-1) kits, serum ammonia by Blood Ammonia assay kit (Njjcbio, A086-1-1)and human albumin (ALB) using Human Albumin ELISA Quantitation Kit (Bethyl, E80-129) according to the manufacturer’s manuals.

**Histological detection of liver injury in mice with ALF**

The liver tissues were evaluated for the degree of liver injury. In brief, the liver tissues were fixed with 4% paraformaldehyde, dehydrated using graded ethanol, and then embedded in paraffin. The paraffin blocks were sectioned and stained with hematoxylin and eosin using standard histological techniques. The necrotic area in injured liver tissues could be distinguished from normal liver tissues by the brighter color and the presence of inflammatory cell infiltration. The liver sections were examined and photo-taken under microscope. The necrotic areas of the injured liver tissues were determined by measuring five independent fields per liver tissue section using ImageJ. The percentage of relative necrotic area (%) were calculated as dividing the necrotic area by total observed area.

**Karyotype analysis**

For the karyotype analysis, exponentially growing HB-orgs at passage 15 were incubated with 50 ng/mL colcemid (Beyotime Biotechnology, ST1173) for 12 hours, then HB-orgs were dissociated into single cells with TrypLE and the chromosomal Giemsa (G)-banding karyotype analysis was performed by Keyida (Guangzhou, China).

**Analysis for liver function**

To determine the secretion levels of ALB and bile acids, the culture medium was collected at indicated time points and analyzed using Human Albumin ELISA Quantitation Kit and Total Bile Acid Detection Kit following manufacturer’s instructions, respectively. For the quantification of urea synthesis, 10 mM ammonium chloride (Sigma, A9434) was supplemented into the culture medium and incubated for 24 hours, then the medium was collected and Urea Assay Kit (Solarbio, BC1535) was used for the quantification according the manufacturer’s protocols. For bile canaliculi assessing, the organoids were incubated with 2 μM CDFDA for 15 minutes at 37 °C, then the organoids were rinsed with PBS for three times and fluorescence images were captured by a single photon confocal microscopy as soon as possible. To assess CYP family genes expression, organoids were incubated with the inducers of rifampicin (Sigma, R3501) at 25 μM or omeprazole (Sigma, O104) at 100 μM for inducing the expressions of CYPs for 48 hours, then qRT‑PCR was performed. To analyze glycogen storge, the organoids were stained with Periodic Acid-Schiff Stain Kit (PAS, Beyotime Biotechnology, C0142S) following manufacturer’s manuals. For the uptake and excretion of indocyanine green (ICG), the organoids were incubated with 1 mg/mL of ICG (MedChemExpress, HY-D0711) for 1 hour in a humidified incubator at 37 °C and 5% CO2, and ICG uptake by the organoids were captured under a microscope, then the organoids were gently washed 3 times with PBS, and fresh medium was supplemented. One hour after the incubation, the excretion of ICG from the organoids was examined under the microscope.

**Oil red O staining**

To assess the formation of lipid droplets, the organoids were fixed with 4% PFA overnight at 4°C after the treatment with FFA at various concentrations for 2 days, then the organoids were rinsed with PBS and stained with Oil red O solution (Sigma, O0625) for 30 minutes at room temperature. The samples were then washed with 60% ethanol for one time and with distilled water three times. Images were obtained by a phase contrast microscope.

**Triglyceride (TG) assay**

For the triglyceride the concentration of the triglyceride, the medium of the organoids treated with FFA at various concentrations were collected and analyzed with a Triglyceride Assay Kit (Njjcbio, A110-1) according to the manufacturer’s protocols.

**Analysis of total RNA sequencing**

Total RNAs of HB-orgs, P-hep-orgs cultured in bioreactor were extracted using RNAiso Plus kit according to the manufacturer’s instructions. The cDNA libraries for RNA sequencing were generated using NEBNext® Ultra™ RNA Library Prep Kit for Illumina (NEB, E7530). Sequencing was performed by Novogene (Beijing, China) on an Illumina HiSeq X-Tensequencer with 150 bp paired- end sequencing reaction. Differentially expressed genes (DEGs) were analyzed by DESeq2 using counts. Genes with P value ≤ 0.05 and |log2 Fold Change| ≥2 was identified as DEGs. Original data were uploaded to the Gene Expression Omnibus database (accession number: GSE265845).

**Single cell RNA sequencing (scRNA-seq) and data analysis**

H9-derived HB-orgs at passage 6 and P-hep-orgs differentiated from HB-orgs at day 8 were dissociated into single cells by TrypLE. BD Rhapsody system was used to capture the transcriptomic information of the single cells. Whole transcriptome libraries were prepared using the BD Rhapsody single-cell whole-transcriptome amplification (WTA) workflow including random priming and extension (RPE), RPE amplification PCR and WTA index PCR. Sequencing was performed by DNBSEQ-T7 (BGISEQ) sequencing platform on a 150 bp paired-end run. The raw digital gene expression matrix was filtered, normalized, and clustered using R and R package Seurat v4.0. Cells with counts fewer than 1000, mitochondrial content more than 40% were removed. Genes detected in less than three cells were removed. The scRNA-seq data of HB-orgs and P-hep-orgs were further integrated with the data of 5-11 weeks human fetal liver cells[3, 4] (data have been deposited to ArrayExpress under accession E-MTAB-7189 and E-MTAB-7407) and 5 independent donor-derived primary human liver cells[5] (GSE115469) respectively, and identified anchors as input for downstream analyses. *t*-SNE mapping was used to visualize scRNA-seq results. The cell type of each cluster was defined based on the results as previously described[5]. The sequencing data from this study have been deposited at the NCBI Gene Expression Omnibus under accession numbers GEO: GSE240097.

**Statistical analysis**

Sample sizes were indicated on the corresponding graph or figure legend; otherwise, n= 3, which represented the number of biological replicates that were analyzed in each experimental group. Data were expressed as the mean ± standard deviation or SEM. Statistical analysis was performed using SPSS. The unpaired, two-tailed Student’s t test and one-way ANOVA (Tukey correction for multiple comparisons) were used to evaluate statistical significance. Differences were considered statistically significant at p-value < 0.05.

**Reference**

1. Sahabian, A., et al., Production and cryopreservation of definitive endoderm from human pluripotent stem cells under defined and scalable culture conditions. Nat Protoc, 2021. **16**(3): p. 1581-1599.

2. He, Z., et al., Liver xeno-repopulation with human hepatocytes in Fah-/-Rag2-/- mice after pharmacological immunosuppression. Am J Pathol, 2010. **177**(3): p. 1311-9.

3. Halpern, K.B., et al., Single-cell spatial reconstruction reveals global division of labour in the mammalian liver. Nature, 2017. **542**(7641): p. 352-356.

4. Wesley, B.T., et al., Single-cell atlas of human liver development reveals pathways directing hepatic cell fates. Nat Cell Biol, 2022. **24**(10): p. 1487-1498.

5. MacParland, S.A., et al., Single cell RNA sequencing of human liver reveals distinct intrahepatic macrophage populations. Nat Commun, 2018. **9**(1): p. 4383.

**Supplementary Figures**


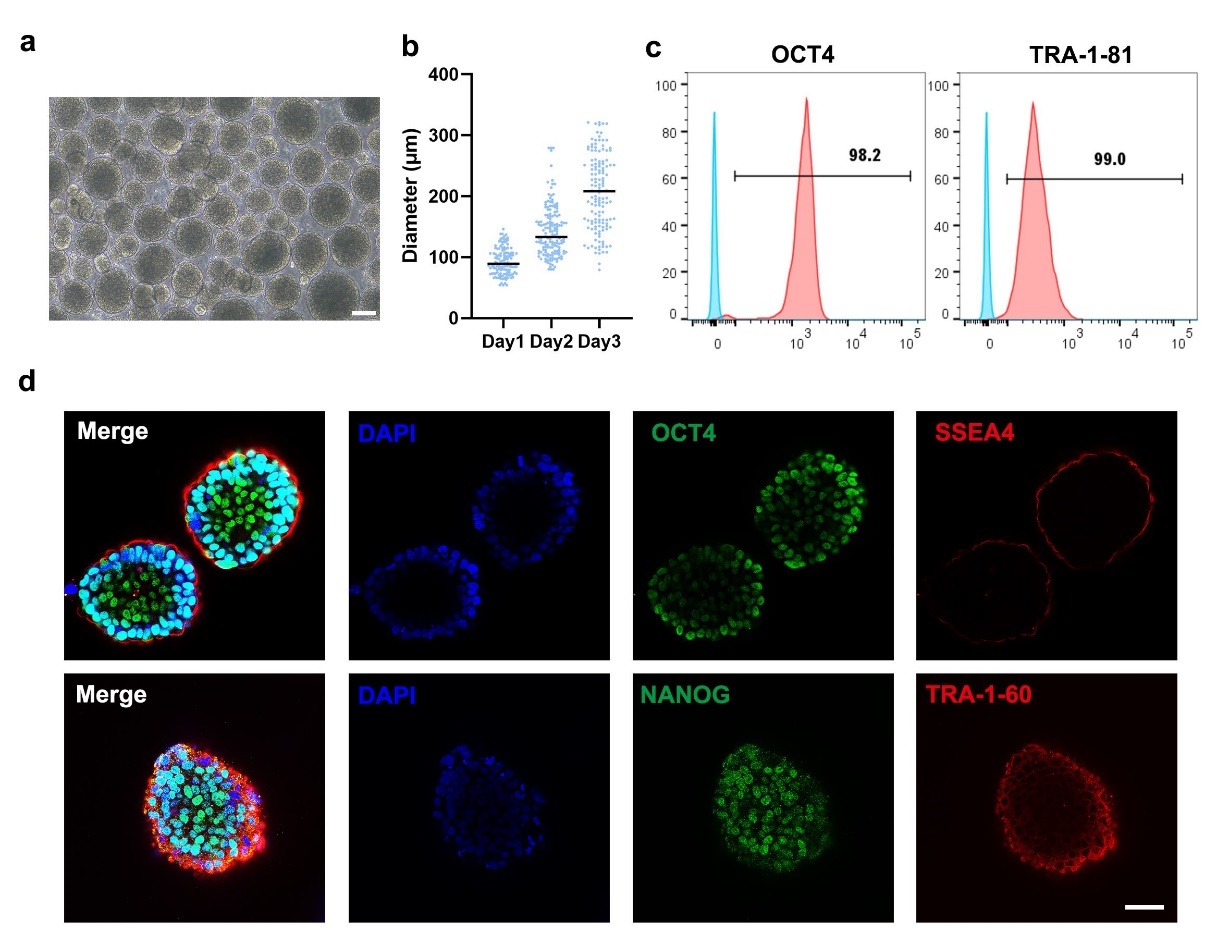


**Figure S1. Characterization of the aggregates of H9 cells under 3D suspension culture condition**

(a) Representative morphology images of the aggregates of H9 cells under 3D suspension culture condition. Scale bar = 100 μm. (b) The average diameters of the aggregates of H9 cells at different days. (c) The percentages of indicated pluripotent markers were measured by flow cytometry. (d) Co-immunostaining for pluripotent markers including OCT4 and SSEA4, NANOG and TRA-1-60 of the aggregates of H9 cells. Nuclei were stained with DAPI (blue). Scale bar = 50 μm.


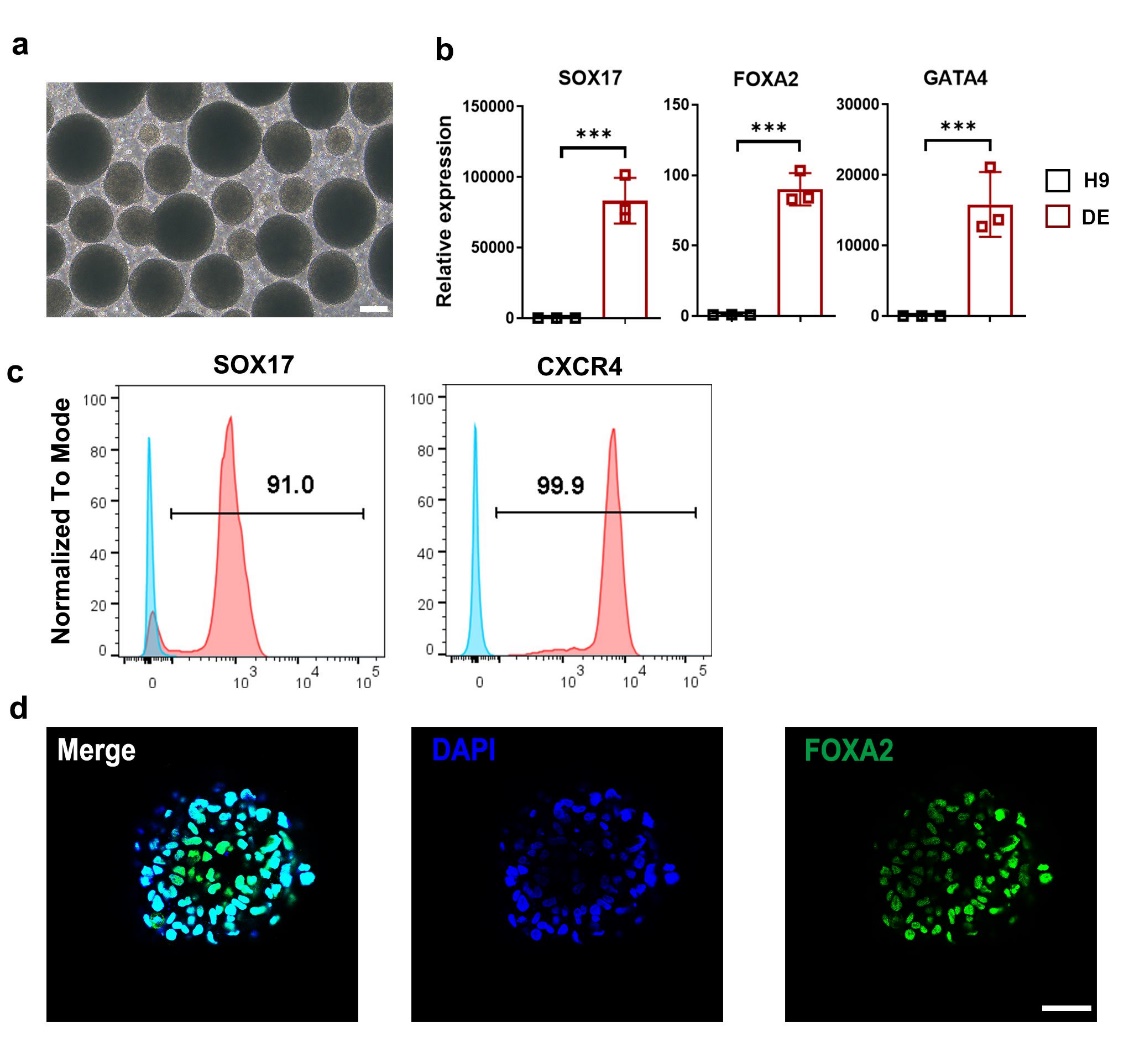


**Figure S2. Characterization of DE spheres induced under 3D suspension culture condition**

(a) Representative morphology image of H9 cell-derived DE spheres induced under 3D suspension culture condition. Scale bar = 100 μm. (b) The relative expression levels of endoderm-related genes in H9 cell-derived DE spheres were analyzed by qRT-PCR (n = 3 for each group). (c) The percentages of positive cells for indicated endoderm markers were measured by flow cytometry. (d) Immunostaining for endoderm marker FOXA2 of H9 cell-derived DE spheres. Nuclei were stained with DAPI (blue). Scale bar = 50 μm. Results were presented as mean ± SD. ****p* <0.001.


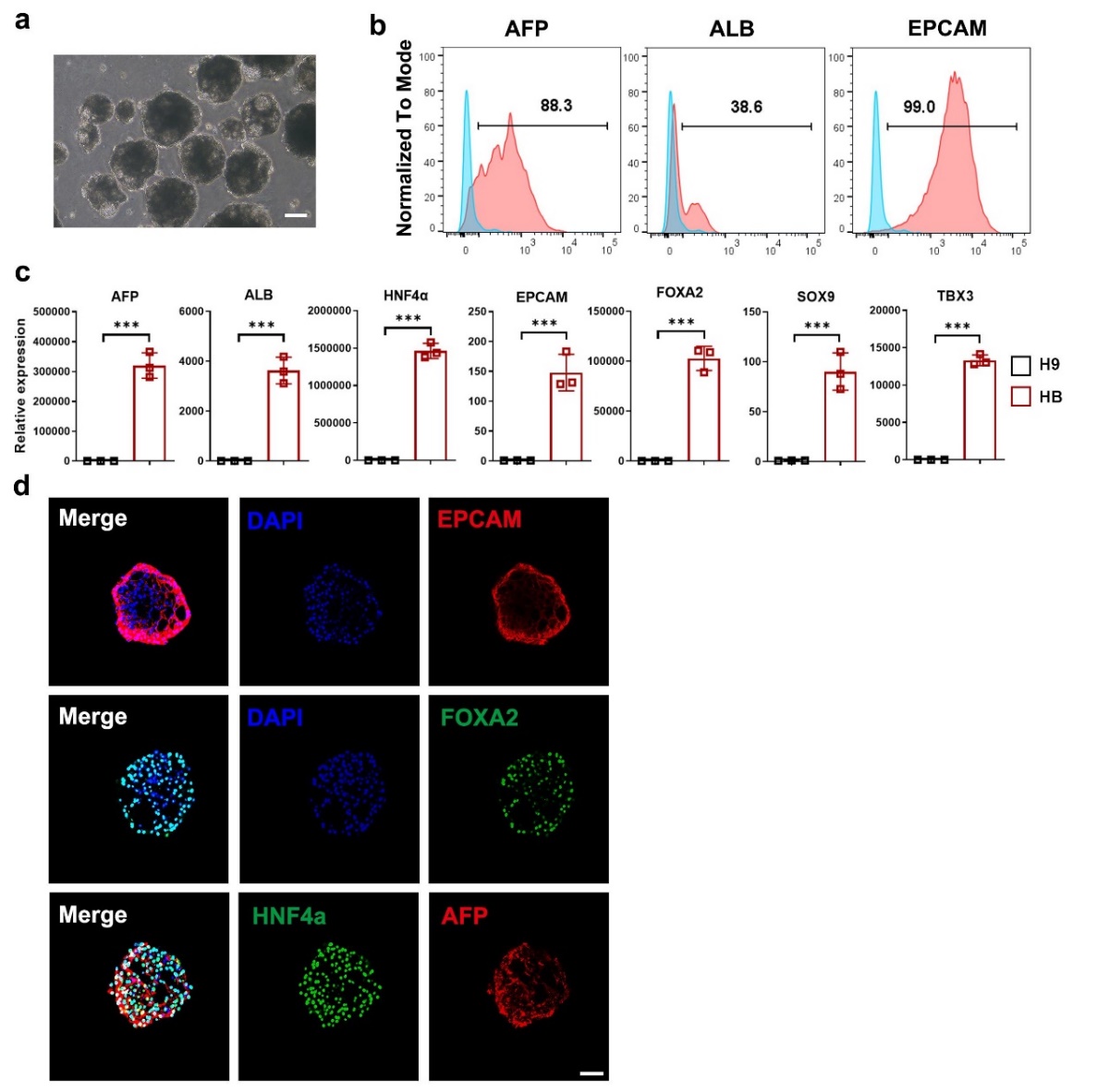


**Figure S3. Characterization of HB spheres generated under 3D suspension culture condition**

(a) Representative morphology image of H9 cell-derived HB spheres generated under 3D suspension culture condition. Scale bar = 100 μm. (b) The percentages of positive cells for indicated hepatic markers were measured by flow cytometry. (c) The relative expression levels of hepatic progenitor cell-related genes in H9 cell-derived HB spheres were determined by qRT-PCR (n = 3 for each group). (d) Immunostaining or co-immunostaining for hepatic progenitor cell markers including EPCAM, FOXA2, HNF4α and AFP in H9 cell-derived HB spheres. Nuclei were stained with DAPI. Scale bar = 100 μm. Results were presented as mean ± SD. ****p* <0.001.


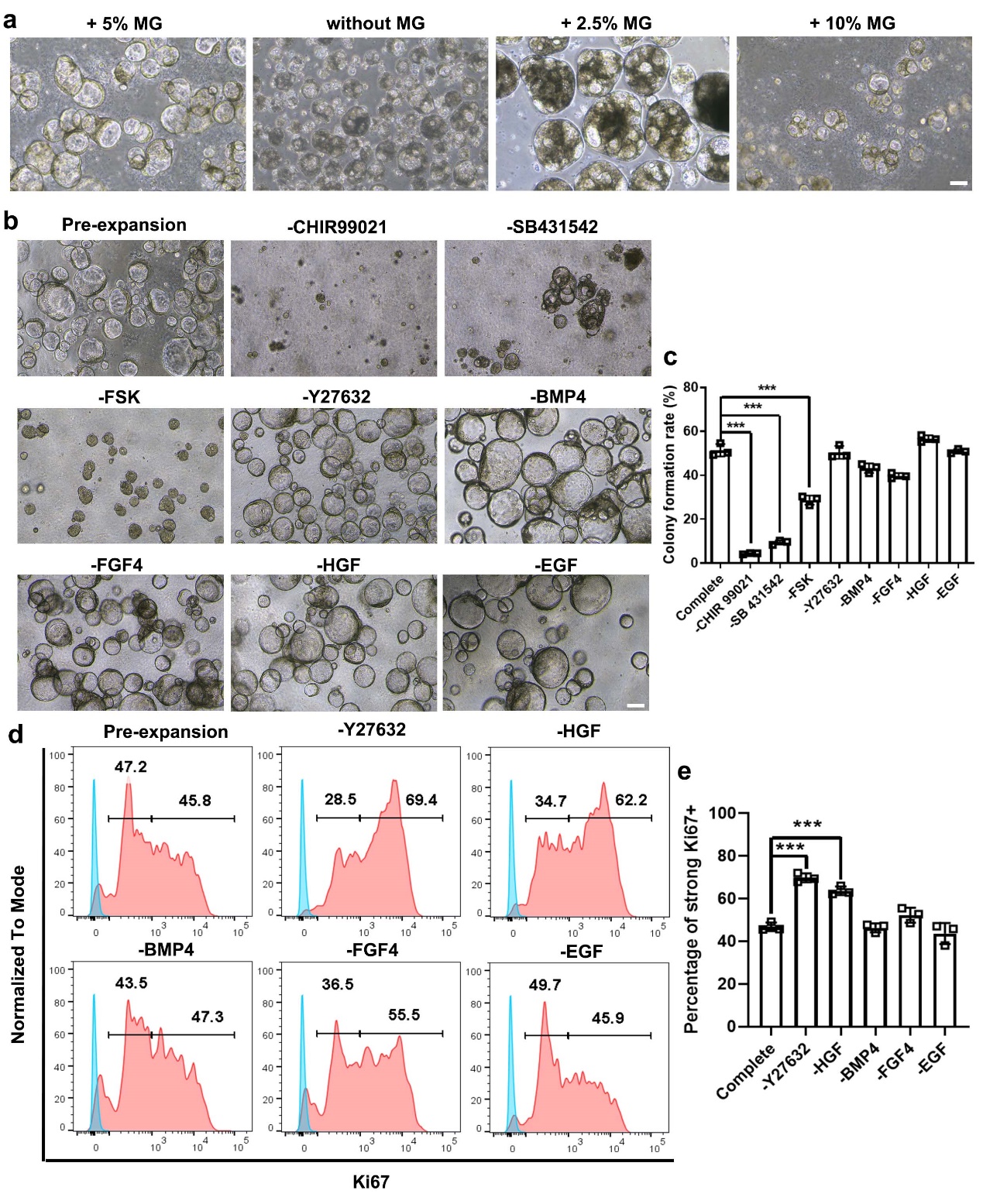


**Figure S4. Development of expansion medium for HB-orgs under 3D suspension culture by removing single chemical compound or growth factor**

(a) Comparing of different concentrations of Matrigel for differentiated hepatoblasts growth. Scale bar = 100 μm. (b) Representative morphology images of HB-orgs under different culture conditions which lacked indicated chemical molecule or growth factor. Scale bar = 100 μm. (c) Quantification of the percentages of colony/organoid formation in each indicated group (n = 3 for each group). (d) The percentages of positive cells for proliferative marker Ki67 were examined by flow cytometry under different culture conditions which lacked indicated chemical molecule or growth factor. (e) The average percentages of Ki67^high^ population in each indicated group were determined by flow cytometry (n = 3 for each group). Results were presented as mean ± SD. ****p* <0.001.


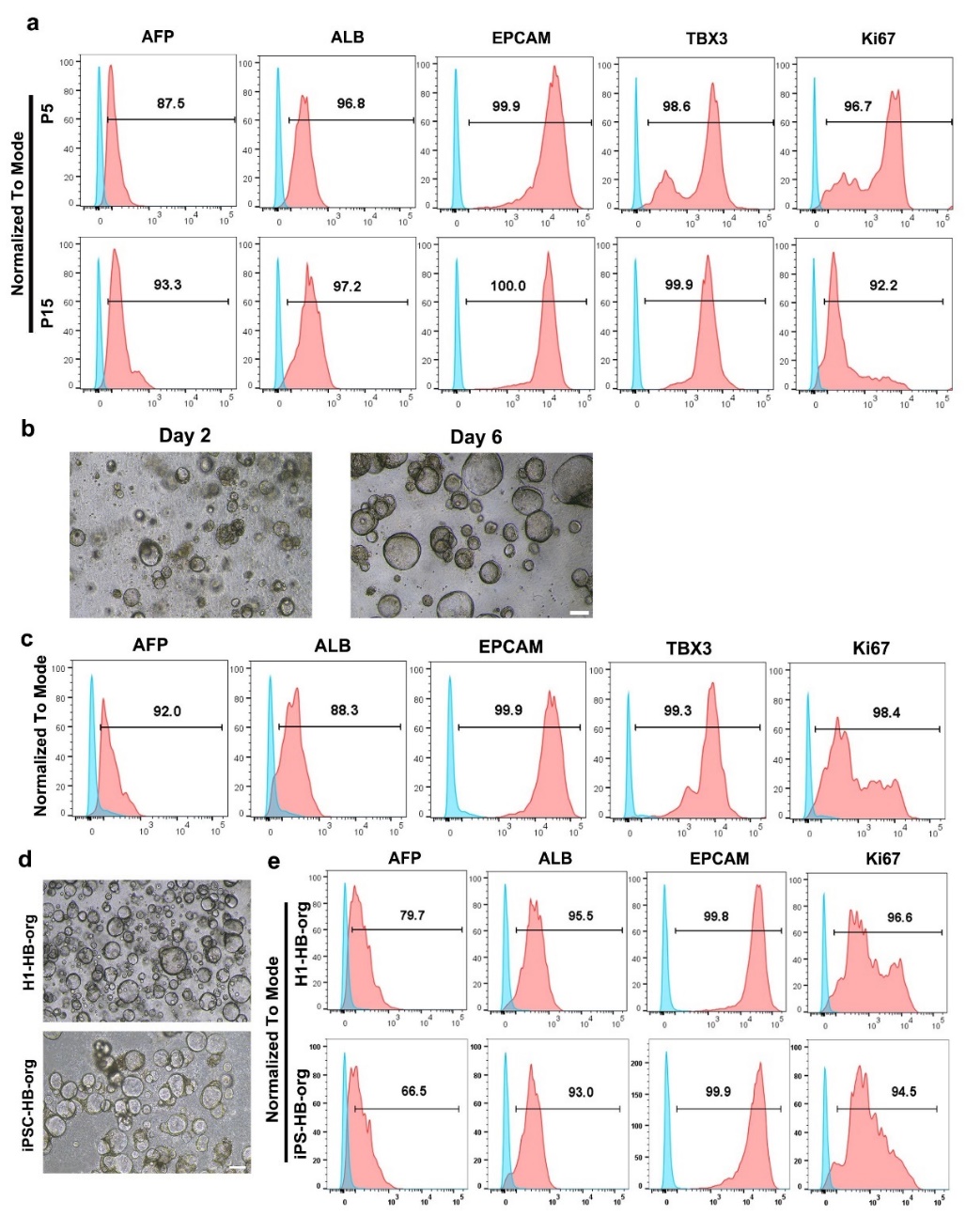


**Figure S5. Characterizations of HB-orgs derived from H9 cells, H1 cells and iPS cells**

1. The percentages of positive cells for HB markers in H9 cell-derived HB-orgs cultured with Matrigel at P5 and P15 were examined by flow cytometry. (b) Representative morphology images of H9 cell-derived HB-orgs at days 2 and 6 post thawing from the cryopreservation. Scale bar = 100 μm. (c) The percentages of positive cells for HB markers after re-culturing H9 cell-derived HB-orgs post thawing were examined by flow cytometry. (d) Representative morphology images of HB-orgs derived from H1 cells or iPS cells at day 5 respectively. Scale bar = 100 μm. (e) The percentages of positive cells for HB markers in HB-orgs derived from H1 cells or iPS cells at P5 were examined by flow cytometry respectively.


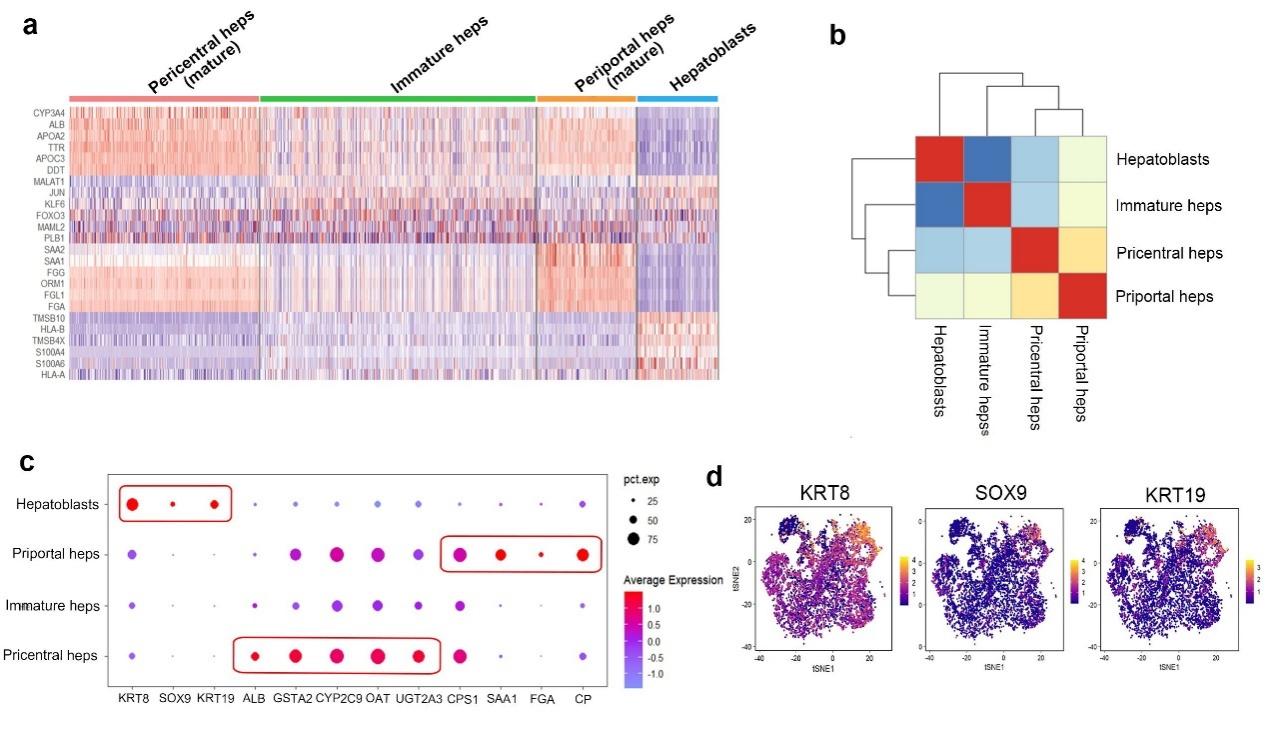


**Figure S6. scRNA-seq profiling of P-hep-orgs**

(a) Heat map showing the expression of signature genes in cell clusters of P-hep-orgs from Fig. 3D. (b) Correlation heatmap of all cell clusters in P-hep-orgs from Fig. 3D. (c) Dot plot representations of marker genes of hepatoblasts (SOX9, KRT19) and pericentral hepatocytes (ALB, GSTA2, CYP2C9, OAT, UGT2A3) as well as periportal hepatocytes (CPS1, SAA1, FGA, CP) in P-hep-orgs. (d) t-SNE representations of hepatoblast marker genes in P-hep-orgs.


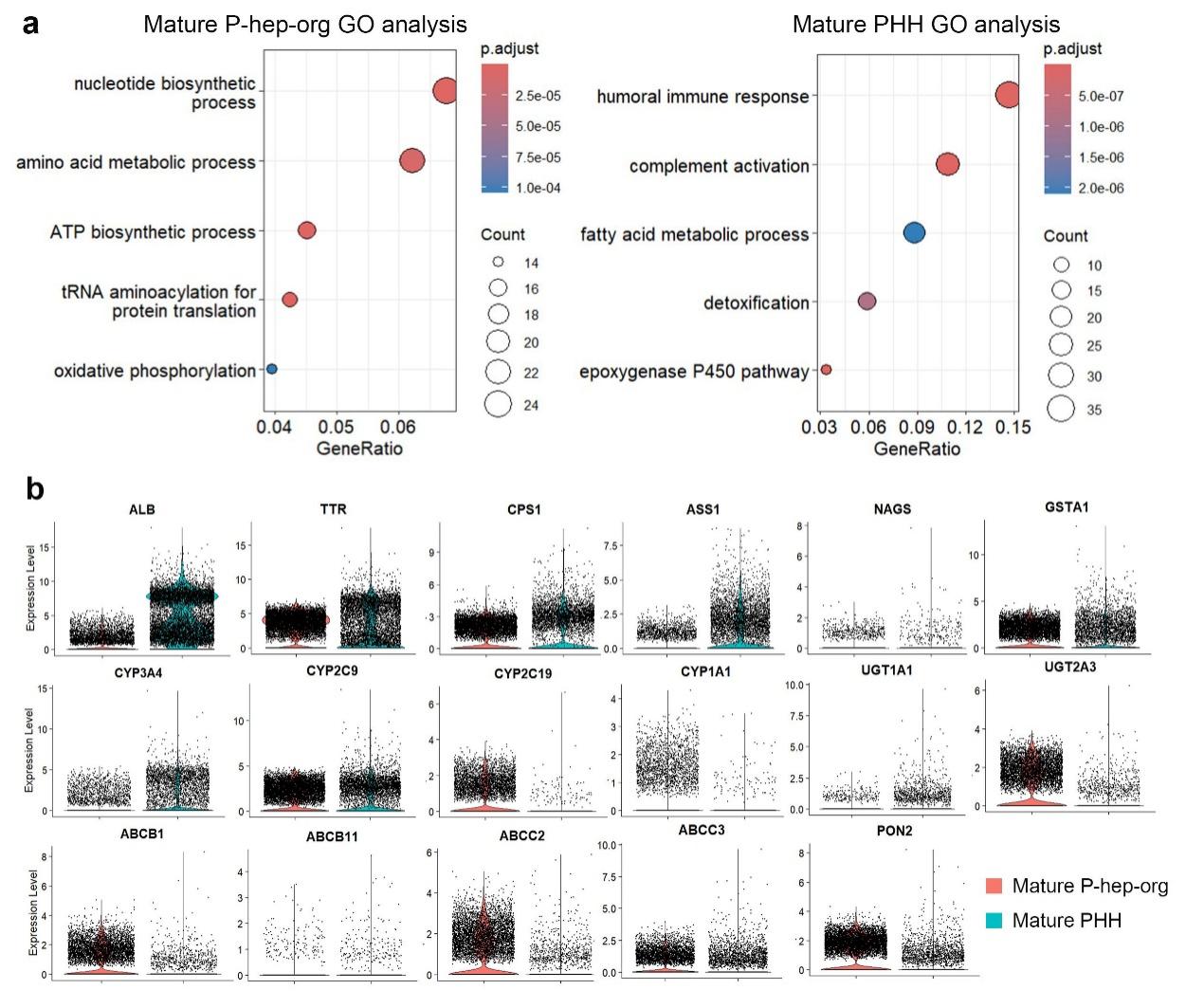


**Figure S7. scRNA-seq profiling of mature hepatocytes from P-hep-orgs and PHHs**

(a) GO analysis of mature P-hep-orgs and PHHs. (b) Violin plot of mature hepatocyte genes in mature P-hep-orgs and PHHs.


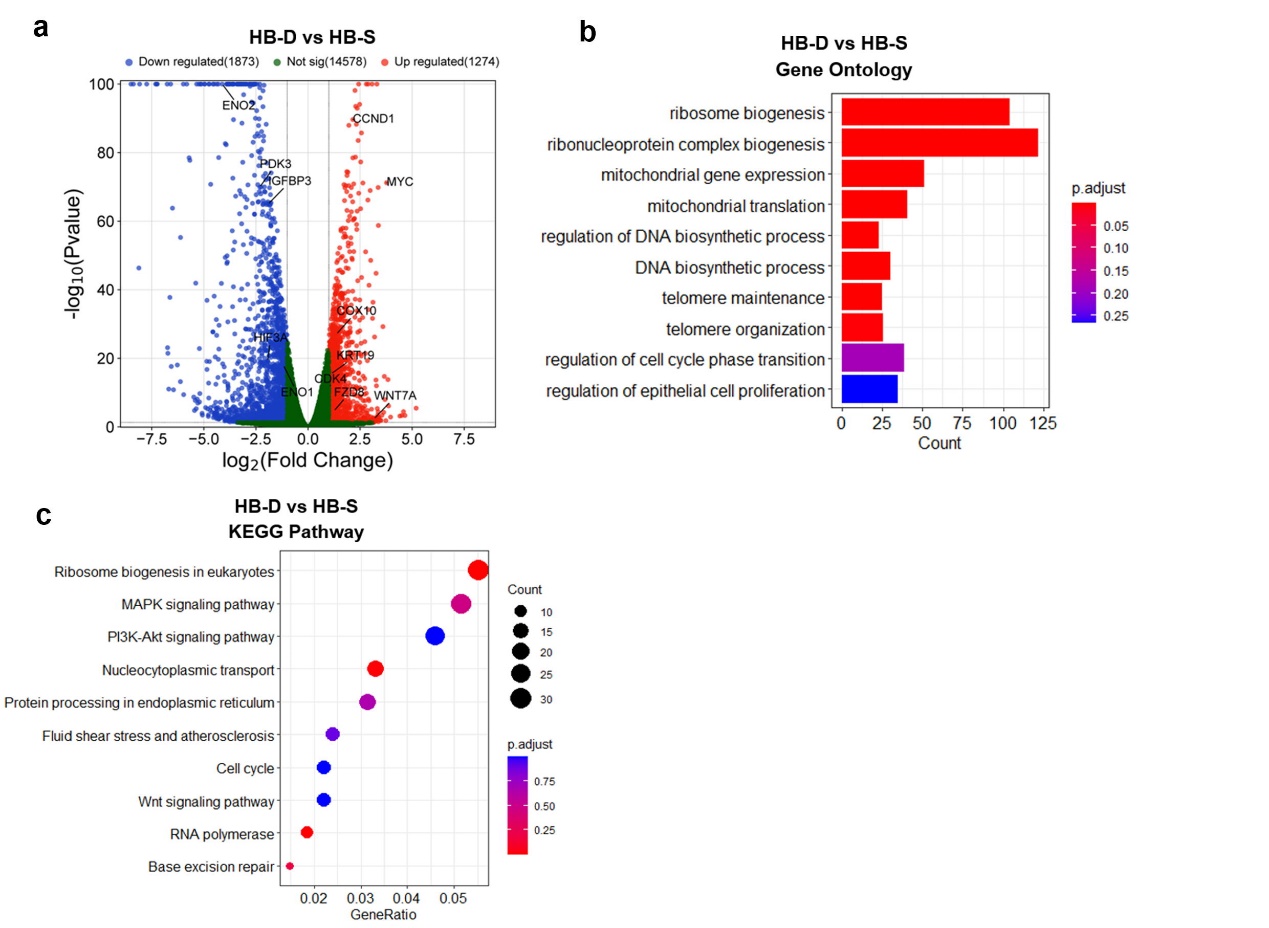


**Figure S8. Transcriptome comparison of HB-orgs cultured under static (HB-S) or dynamic (HB-D) suspension conditions**

(a) Volcano plot showing transcriptome changes between HB-D and HB-S. (b, c) Enriched Go terms (b) and KEGG pathways (c) analysis of upregulated genes in HB-D.


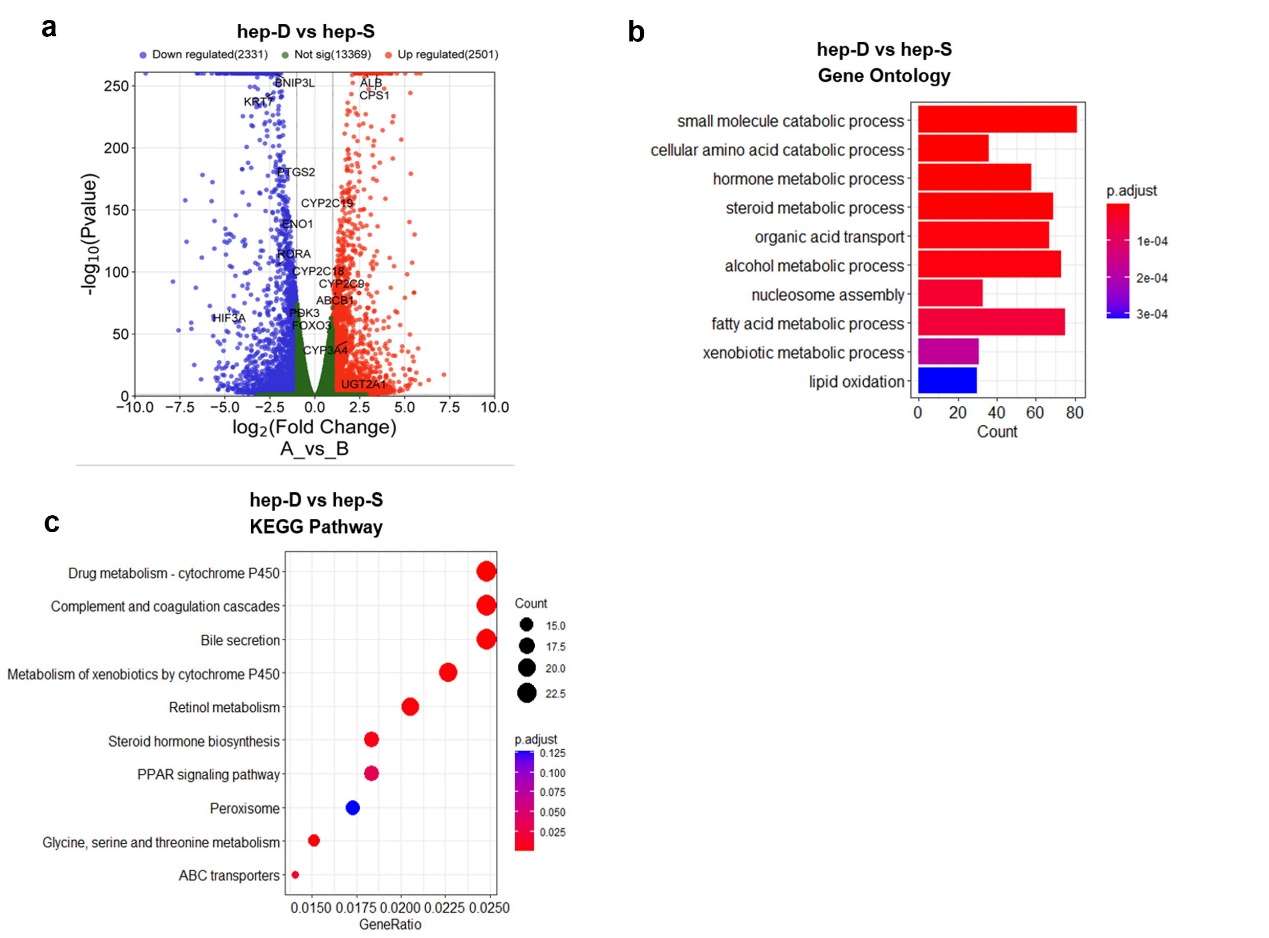


**Figure S9. Transcriptome comparison of P-hep-orgs cultured under static (hep-S) or dynamic (hep-D)** **suspension conditions**

(a) Volcano plot showing transcriptome changes between hep-D and hep-S. (b, c) Enriched Go terms (b) and KEGG pathways (c) analysis of upregulated genes in hep-D.

**Table S1 Primers used in this study**

| Gene | Forward | Reverse |
| --- | --- | --- |
| GAPDH | GAAGATGGTGATGGGATTTC | GAAGGTGAAGGTCGGAGTC |
| FoxA2 | GACAAGTGAGAGAGCAAGTG | ACAGTAGTGGAAACCGGAG |
| SOX17 | CGAGTTGAGCAAGATGCTGG | TTGTAGTTGGGGTGGTCCTG |
| GATA4 | GCGGTGCTTCCAGCAACTCCA | GACATCGCACTGACTGAGAACG |
| AFP | GGGAGCGGCTGACATTAT | TGTTTCATCCACCACCAA |
| ALB | CTGCCTGCCTGTTGCCAAAGC | GGCAAGGTCCGCCCTGTCATC |
| HNF4a | GGTGTCCATACGCATCCTTGAC | AGCCGCTTGATCTTCCCTGGAT |
| CK19 | ACCAAGTTTGAGACGGAACAG | CCCTCAGCGTACTGATTTCCT |
| SOX9 | AGCGAACGCACATCAAGAC | CTGTAGGCGATCTGTTGGGG |
| TBX3 | GGATGTCCAAAGTCGTCA | GCTGGTATTTGTGCATGGAGTTCA |
| EPCAM | AATCGTCAATGCCAGTGTACTT | TCTCATCGCAGTCAGGATCATAA |
| Ki67 | ACGCCTGGTTACTATCAAAAGG | CAGACCCATTTACTTGTGTTGGA |
| α1AT | TCGCTACAGCCTTTGCAATG | TTGAGGGTACGGAGGAGTTCC |
| FASN | TTCTACGGCTCCACGCTCTTCC | GAAGAGTCTTCGTCAGCCAGGA |
| CIDEA | TGACATTCATGGGATTGCAGAC | CATGGTTTGAAACTCGAAAAGGG |
| DGAT1 | AATAAAGGATCTGCCCTGTCACG | TTGAGCCAGGTGACAGAGAAGATG |
| PLIN2 | CTTGTGTCCTCCGCTTATGTC | GCAGAGGTCACGGTCTTCAC |
| PNPLA2 | ATGTTCCCGAGGGAGACCAA | GAGGCTCCGTAGATGTGAGTG |
| SOX17 | GTGGACCGCACGGAATTTG | GAGGCCCATCTCAGGCTTG |
| FOXA2 | GGAACACCACTACGCCTTCAAC | AGTGCATCACCTGTTCGTAGGC |
| GATA4 | CGACTTCTCAGAAGGCAGAGAGTG | CTTCATGTAGAGGCCGCAGGCATT |
| AE2 | GACTTCCGAGATGCACTTGACC | CCTATCAGGTCCTGCGTCTTCT |
| AQP1 | TATGCGTGCTGGCTACTACCGA | GGTTAATCCCACAGCCAGTGTAG |
| HES1 | GGAAATGACAGTGAAGCACCTCC | GAAGCGGGTCACCTCGTTCATG |
| FABP6 | AGTTCACTGTTGGCAAGGAAAGC | CACGATCTCTGAGGTCTGGTGA |
| ASBT | CTGGTTGCTCTCGTTGTTCCTG | TCCTCCAACCACAGCTATGAGC |
| GGT1 | TGACGTACCACCGCATCGTAGA | CAGCGAAGAACTCGGAGGTCAT |
| CFTR | GGAGAGCATACCAGCAGTGACT | TTCCAAGGAGCCACAGCACAAC |
| MPP3 | GCTGCTGACTTACGAAGAGGTG | CCACCTTTTGCTTCAGCTCGTG |
| CK7 | TCCGCGAGGTCACCATTAAC | GCTCTGTCAACTCCGTCTCAT |

**Table S2 Antibodies used in this study**

| Primary antibody | | | | |
| --- | --- | --- | --- | --- |
| Antibody | Company | Product code | Ig Species | Dilution |
| Anti-GAPDH | Abcam | ab128915 | Rabbit | 10000 |
| Anti-Keratin19 | CST | 12434 | Rabbit | 50 |
| Anti-Ki67 | CST | 9449 | Mouse | 800 |
| Anti-SOX9 | CST | 82630 | Rabbit | 500 |
| Anti-EPCAM | CST | 2929 | Mouse | 800 |
| Anti-HNF4a | CST | 3113 | Rabbit | 2000 |
| Anti-ALB | Bethyl | A80-129A | Goat | 1000 |
| Anti-E-Cadherin | CST | 3195 | Rabbit | 200 |
| Anti-AFP | Thermo | MIA1305 | Mouse | 200 |
| Anti-Keratin18 | CST | 4546 | Mouse | 200 |
| Anti-a1AT | Bethyl | A80-122A | Goat | 200 |
| Anti-ASGPR | Santacruz | sc-52623 | Mouse | 100 |
| Anti-ZO1 | CST | 13663 | Rabbit | 200 |
| Anti-MDR1 | CST | 13342 | Rabbit | 200 |
| Anti-SLC10A1(NTCP) | Boster | PB9745 | Rabbit | 400 |
| Anti-OCT4 | CST | 2750 | Rabbit | 200 |
| Anti-SSEA4 | CST | 4755 | Mouse | 500 |
| Anti-NANOG | CST | 4903 | Rabbit | 200 |
| Anti-TRA-1-60 | CST | 4746 | Mouse | 1000 |
| Anti-FOXA2 | CST | 8186 | Rabbit | 400 |
| Anti-CK7 | CST | 4465 | Rabbit | 200 |
| PE anti-human TRA-1-81 Antibody | Biolegend | 330708 |  | 5 μL for million cells |
| PE Mouse anti-Human Sox17 Antibody | Biolegend | 561591 |  | 5 μL for million cells |
| APC/Cyanine7 anti-human CD184 (CXCR4) Antibody | Biolegend | 306528 |  | 5 μL for million cells |
| PE Mouse Anti-Human Alpha-fetoprotein Antibody | BD | 563002 |  | 5 μL for million cells |
| Human Serum Albumin PE-conjugated Antibody | R&D | IC1455P |  | 10 μL for million cells |
| PE anti-human CD326 (Ep-CAM) | Biolegend | 324206 |  | 5 μL for million cells |
| Rabbit Anti-TBX3 antibody | Bioss | bs-10266R |  | 3ug/test |
| PE anti-human ASGPR1 antibody | BD | 563655 |  | 5 μL for million cells |
| Goat anti-Human-A1AT（FITC） antibody | Bethyl | A80-122F |  | 1000 |
| PE mouse isotype-controlled antibody | BD | 556650 |  | 20 μL for million cells |
| APC mouse isotype-controlled antibody | BD | 555751 |  | 20 μL for million cells |
| Anti-Factin (Phalloidin-iFluor 594) | Abcam | ab176757 |  |  |
|  |  |  |  |  |
| Secondary antibody | Company | Code number | Dilution |  |
| Alexa Fluor 594-conjugated goat anti-Rabbit IgG | CST | 8889 | 800 |  |
| Alexa Fluor 488-conjugated goat anti-Rabbit IgG | CST | 4412 | 800 |  |
| Alexa Fluor 594-conjugated goat anti-Mouse IgG | CST | 8890 | 800 |  |
| Alexa Fluor 488-conjugated goat anti-Mouse IgG | CST | 4408 | 800 |  |
| Alexa Fluor 594-conjugated donkey anti-Goat IgG | Abcam | ab150136 | 800 |  |
